# Supplementary material for: Relationship between Green and Blue Spaces with Mental and Physical Health: A Systematic Review of Longitudinal Observational Studies
Source: Int J Environ Res Public Health. 2021 Aug 26;18(17):9010. doi: 10.3390/ijerph18179010 (PMC8431638; doi:10.3390/ijerph18179010)
Supplement: Supplementary file 1 [file ijerph-18-09010-s001.zip › ijerph-1307799-supplementary/Supplementary Material S1.pdf]

MEDLINE via Ovid

1 exp Depression/
118775

2 exp Anxiety Disorders/
79201

3 exp Obsessive-Compulsive Disorder/
14545

4 exp Stress Disorders, Post-Traumatic/
32558

5 exp Psychotic Disorders/
52066

6 exp Bipolar Disorder/
40222

7 exp Schizophrenia/ 1
04398

8 (depression or depressive or dysthymi* or anxiety or (anxiety adj disorder*) or (panic adj disorder*) or (generali?ed adj anxiety adj disorder*) or ocd or (obsessive adj compulsive) or obsessive-compulsive or ptsd or posttrauma* or (post adj trauma* adj disorder*) or bipolar or (bipolar and (affective or disorder*)) or schizophreni* or psychosis).ti,ab,kw.
692588

9 1 or 2 or 3 or 4 or 5 or 6 or 7 or 8 / 792883

10 physical health.ti,ab,kw.
20946

11 (cardio-vascular or cardiovascular or (cardio adj vascular) or myocardial).ti,ab,kw.
723463

12. cancer.ti,ab,kw.
1697426

13 (obesity or BMI or (body adj mass) or diabetes).ti,ab,kw.
872407

14 (respiratory or cardio-respiratory or cardiorespiratory or (cardio adj respiratory)).ti,ab,kw.
444098

15 Endocrine ti,ab,kw.
121939

16 Musculoskeletal Diseases/
12705

17 (musculoskeletal or musculo-skeletal).ti,ab,kw.
51498

18 exp Cardiovascular Diseases/
2380012

19 neoplasms/ or musculoskeletal diseases/ or digestive system diseases/ or respiratory tract diseases/ or nervous system diseases/ or eye diseases/ or "skin and connective tissue diseases"/ or "nutritional and metabolic diseases"/ or endocrine system diseases/ or immune system diseases/ or "disorders of environmental origin"/ or occupational diseases/
632776

20 (physical adj function*).ti,ab,kw.
24092

21 "Quality of Life"/
194476

22 ((quality adj of adj life) or insomnia or sleep or (sleep adj disrupt*)).ti,ab,kw.
439635

23 Frailty/
2627

24
(frail or frailty).ti,ab,kw.
21871

25 10 or 11 or 12 or 13 or 14 or 15 or 16 or 17 or 18 or 19 or 20 or 21 or 22 or 23 or 24
6104617

26 (health adj promot* adj behavio?r*).ti,ab,kw.
1454

27 health promot* behavio?r*.ti,ab,kw.
1417

28 Diet/
159149

29 exp Exercise/
194919

30
exp Smoking/
147018

31 exp Alcohol Drinking/
68918

32 ((physical* adj activ*) or (physical adj activity) or walking or running).ti,ab,kw.
233038

33 ((alcohol adj drinking) or (alcohol adj consumption) or smoking or diet).ti,ab,kw.
568833

34 26 or 27 or 28 or 29 or 30 or 31 or 32 or 33
1041527

35 9 or 25 or 34
7332636

36 *Environment/ or *Environment Design/
29764

37 (green adj2 (area$ or cover or environment$ or gym$ or neighbourhood$ or neighborhood$ or roadside$ or space$)).ti,ab,kw.
2444

38 ((city or cities or environment$ or neighbourhood or neighborhood or urban) adj2 greening).ti,ab,kw.
104

39 ((ambient or city or cities or environment$ or neighbourhood or neighborhood or residential or surrounding or urban) adj2 greenness).ti,ab,kw.
186

40 (greenery or greenspace* or greenness).ti,ab,kw.
1246

41 (garden$ or park or parks).ti,ab,kw.
31889

42 sports field$.ti,ab,kw.
99

43 wilderness area$.ti,ab,kw.
179

44 public open space$.ti,ab,kw.
99

45 neighbourhood open space$.ti,ab,kw.
2

46 neighborhood open space$.ti,ab,kw.
4

47 36 or 37 or 38 or 39 or 40 or 41 or 42 or 43 or 44 or 45 or 46
63889

48 *Residence Characteristics/
13633

49 47 or 48
76049

50 *Water/
61472

51 *Rivers/
12620

52 *"Oceans and Seas"/
1126

53 (bluespace$ or blue space$).ti,ab,kw.
114

54 bluehealth.ti,ab,kw.
1

55 blue water$.ti,ab,kw.
190

56 blue gym$.ti,ab,kw.
3

57 50 or 51 or 52 or 53 or 54 or 55 or 56
75208

58 *air pollution/
22636

59 *air quality/
22636

60 (air adj2 (quality or pollution)).ti,ab,kw.
32154

61 ((ambient or neighbourhood or neighborhood or outdoor) adj2 air).ti,ab,kw.
12961

62 58 or 59 or 60 or 61
51241

63 exp noise/
23996

64 *noise pollution/
12458

65 ((traffic or aircraft or industr$ or neighbourhood or neighborhood or outdoor) adj2 noise).ti,ab,kw.
2190

66 (noise adj2 pollution).ti,ab,kw.
832

67 63 or 64 or 65 or 66
24736

68 *Nature/
546

69 ((natural or outdoor$ or salutogenic) adj2 environment$).ti,ab,kw.
14691

70 ((nature or natural) adj2 space$).ti,ab,kw.
396

71 68 or 69 or 70
15550

72 49 or 57 or 62 or 67 or 71
237726

73 35 and 72
43562

74 exp animals/ not humans.sh.
4718023

75 73 not 74
38821

76 exp cohort studies/
2010640

77 ((cohort or longitudinal or follow-up or prospective or retrospective) adj2 stud*).ti,ab.
782804

78 (cohort or follow-up or longitudinal).ti,ab.
1565994

79 longitudinal study/
135701

80 76 or 77 or 78 or 79
2909197

81 (health adj promot$ adj2 environment$).ti,ab,kw.
247

82<br>39051	75 or 81
83<br>6606	80 and 82

EMBASE via Ovid

1 exp depression/
472047

2 exp anxiety/
208150

3 exp anxiety disorder/
240819

4 exp obsessive compulsive disorder/
39417

5 exp posttraumatic stress disorder/
59929

6 exp psychosis/
277139

7 exp bipolar disorder/ or bipolar depression/ or bipolar II disorder/ or bipolar I disorder/
62961

8 exp schizophrenia/
177844

9 (depression or depressive or dysthymi* or anxiety or (anxiety adj disorder*) or (panic adj disorder*) or (generali?ed adj anxiety adj disorder*) or ocd or (obsessive adj compulsive) or obsessive-compulsive or ptsd or posttrauma* or (post adj trauma* adj disorder*) or bipolar or (bipolar and (affective or disorder*)) or schizophreni* or psychosis).ti,ab,kw.
938420

10 1 or 2 or 3 or 4 or 5 or 6 or 7 or 8 or 9
1218837

11 physical health.ti,ab,kw.
27892

12 (cardio-vascular or cardiovascular or (cardio adj vascular) or myocardial).ti,ab,kw.
1054449

13 cancer.ti,ab,kw.
2447213

14 (obesity or BMI or (body adj mass) or diabetes).ti,ab,kw.
1357330

15 (respiratory or cardio-respiratory or cardiorespiratory or (cardio adj respiratory)).ti,ab,kw.
613602

16 endocrine.ti,ab,kw.
175359

17 exp musculoskeletal disease/
2218427

18 (musculoskeletal or musculo-skeletal).ti,ab,kw.
74352

19 cardiovascular risk/ or exp cardiovascular disease/
4070078

20 exp malignant neoplasm/
3427572

21 exp digestive system disease/
3092666

22 exp respiratory tract disease/
2411729

23 exp neurologic disease/
3451283

24 exp eye disease/
906215

25 skin disease/
66380

26 metabolic disorder/
64409

27 exp diabetes mellitus/
952361

28 exp obesity/
516495

29 exp endocrine disease/
2002252

30 immunopathology/
26115

31 exp occupational disease/
128603

32 exp environmental disease/
1414

33 (neoplasm* or musculoskeletal disease* or digestive system or respiratory tract diseases* or nervous system or eye disease* or "skin and connective tissue diseases" or "nutritional and metabolic diseases" or endocrine system disease* or immune system disease* or environmental disease* or HIV or human immunodeficiency or occupational disease or diabetes or diabetic or myocardial).ti,ab,kw.
2376383

34 exp rare disease/
37860

35 11 or 12 or 13 or 14 or 15 or 16 or 17 or 18 or 19 or 20 or 21 or 22 or 23 or 24 or 25 or 26 or 27 or 28 or 29 or 30 or 31 or 32 or 33 or 34
15877053

36 (physical adj function*).ti,ab,kw.
38340

37 "quality of life"/
469182

38 (quality of life or insomnia or sleep or sleep disrupt*).ti,ab,kw.
694792

39 insomnia/
66505

40 exp frailty/
11054

41 (frail or frailty).ti,ab,kw.
34138

42 (health adj promot* adj behavio?r*).ti,ab,kw.
1559

43 health promot* behavio?r*.ti,ab,kw.
1559

44 diet/
216331

45 exercise/ or "physical activity, capacity and performance"/
275876

46 exp physical activity/
418363

47 exp smoking/
385634

48 exp alcohol consumption/
127555

49 ((physical* adj activ*) or (physical adj activity) or walking or running).ti,ab,kw.
316971

50 ((alcohol adj drinking) or (alcohol adj consumption) or smoking or diet).ti,ab,kw.
780403

51 36 or 37 or 38 or 39 or 40 or 41 or 42 or 43 or 44 or 45 or 46 or 47 or 48 or 49 or 50
2489556

52 10 or 35 or 51
17346597

53 (green adj2 (area$ or cover or environment$ or gym$ or neighbourhood$ or neighborhood$ or roadside$ or space$)).ti,ab,kw.
2828

54 ((city or cities or environment$ or neighbourhood or neighborhood or urban) adj2 greening).ti,ab,kw.
132

55 ((ambient or city or cities or environment$ or neighbourhood or neighborhood or residential or surrounding or urban) adj2 greenness).ti,ab,kw.
213

56 (greenery or greenspace* or greenness).ti,ab,kw.
1314

57 (garden$ or park or parks).ti,ab,kw.
38736

58 sports field$.ti,ab,kw.
140

59 wilderness area$.ti,ab,kw.
220

60 public open space$.ti,ab,kw.
116

61 neighbourhood open space$.ti,ab,kw.
2

62 neighborhood open space$.ti,ab,kw.
3

63 (bluespace$ or blue space$).ti,ab,kw.
117

64 bluehealth.ti,ab,kw.
1

65 blue water$.ti,ab,kw.
225

66 blue gym$.ti,ab,kw.
3

67 air pollution/
60231

68 air quality/
16896

69 (air adj2 (quality or pollution)).ti,ab,kw.
50338

70 ((ambient or neighbourhood or neighborhood or outdoor) adj2 air).ti,ab,kw.
17790

71 exp noise/
108517

72 exp noise pollution/
8939

73 ((traffic or aircraft or industr$ or neighbourhood or neighborhood or outdoor) adj2 noise).ti,ab,kw.
2870

74 (noise adj2 pollution).ti,ab,kw.
1165

75 ((natural or outdoor$) adj2 environment$).ti,ab,kw.
16533

76 ((nature or natural) adj2 space$).ti,ab,kw.
417

77 exp cohort analysis/
594604

78 longitudinal study/
141374

79 ((cohort or longitudinal or follow-up or prospective or retrospective) adj2 stud*).ti,ab.
1156722

80 (cohort or follow-up or longitudinal).ti,ab.
2450549

81 77 or 78 or 79 or 80
3045207

82 (health adj promot$ adj2 environment$).ti,ab,kw.
290

83 53 or 54 or 55 or 56 or 57 or 58 or 59 or 60 or 61 or 62 or 63 or 64 or 65 or 66 or 67 or 68 or 69 or 70 or 71 or 72 or 73 or 74 or 75 or 76
262719

84 52 and 83
85760

85 82 or 84
86038

86 81 and 85
10247

PsycINFO via Ovid

1 exp "Long-term Depression (Neuronal)"/ or exp Spreading Depression/ or exp "Depression (Emotion)"/ or exp Major Depression/ or exp Reactive Depression/ or exp Beck Depression Inventory/ or exp Treatment Resistant Depression/ or exp Postpartum Depression/ or exp Atypical Depression/ or exp Endogenous Depression/ or exp Anaclitic Depression/ or exp Recurrent Depression/ or exp Late Life Depression/
157037

2 exp Anxiety Disorders/
53997

3 exp Anxiety/
71596

4 exp Panic/ or exp Panic Disorder/
9213

5 exp Obsessive Compulsive Disorder/
15127

6 exp Posttraumatic Stress Disorder/
33049

7 exp Schizophrenia/ or exp Diagnosis/ or exp Psychosis/
315653

8 exp Bipolar Disorder/
30512

9 (depression or depressive or dysthymi* or anxiety or (anxiety adj disorder*) or (panic adj disorder*) or (generali?ed adj anxiety adj disorder*) or ocd or (obsessive adj compulsive) or obsessive-compulsive or ptsd or posttrauma* or (post adj trauma* adj disorder*) or bipolar or (bipolar and (affective or disorder*)) or schizophreni* or psychosis).ti,ab.
570777

10 1 or 2 or 3 or 4 or 5 or 6 or 7 or 8 or 9
773959

11 physical health.ti,ab.
19009

12 (cardio-vascular or cardiovascular or (cardio adj vascular) or myocardial).ti,ab.
32556

13 cancer.ti,ab.
59494

14 (obesity or BMI or (body adj mass) or diabetes or diabetic).ti,ab.
77289

15 (respiratory or cardio-respiratory or cardiorespiratory or (cardio adj respiratory)).ti,ab.
17587

16 endocrine.ti,ab.
8450

17 exp Musculoskeletal Disorders/
18258

18 (musculoskeletal or musculo-skeletal).ti,ab.
5586

19 exp Cardiovascular Disorders/
62101

20 exp Neoplasms/
51916

21 exp Digestive System Disorders/
13924

22 exp Respiratory Tract Disorders/
14722

23 exp Nervous System/
366236

24 exp Eye Disorders/
4837

25 exp Metabolic Rates/
1141

26 exp Endocrine Disorders/
22096

27 exp Immune System/
4795

28 exp HIV/ or exp Immune System/
47620

29 exp Occupational Health/
3974

30 (neoplasm* or musculoskeletal disease* or digestive system or respiratory tract diseases* or nervous system or eye disease* or "skin and connective tissue diseases" or "nutritional and metabolic diseases" or endocrine system disease* or immune system disease* or environmental disease* or HIV or human immunodeficiency or occupational disease or diabetes or diabetic or myocardial).ti,ab.
132516

31 11 or 12 or 13 or 14 or 15 or 16 or 17 or 18 or 19 or 20 or 21 or 22 or 23 or 24 or 25 or 26 or 27 or 28 or 29 or 30
708983

32 (physical adj function*).ti,ab.
6250

33 exp "Quality of Life"/
43074

34 ((quality adj of adj life) or insomnia or sleep or (sleep adj disrupt*)).ti,ab.
135559

35 (frail or frailty).ti,ab.
4367

36 (health adj promot* adj behavio?r*).ti,ab.
1036

37 health promot* behavio?r*.ti,ab.
1036

38 exp Diets/
17057

39 exp Exercise/
26402

40 exp Physical Activity/
41965

41 exp Tobacco Smoking/
32604

42 exp alcoholism/
30678

43 ((physical* adj activ*) or (physical adj activity) or walking or running).ti,ab.
62325

44 ((alcohol adj drinking) or (alcohol adj consumption) or smoking or diet).ti,ab.
87131

45 32 or 33 or 34 or 35 or 36 or 37 or 38 or 39 or 40 or 41 or 42 or 43 or 44
337589

46 10 or 31 or 45
1489329

47 environment/
17774

48 (green adj2 (area$ or cover or environment$ or gym$ or neighbourhood$ or neighborhood$ or roadside$ or space$)).ti,ab.
555

49 ((city or cities or environment$ or neighbourhood or neighborhood or urban) adj2 greening).ti,ab.
16

50 ((ambient or city or cities or environment$ or neighbourhood or neighborhood or residential or surrounding or urban) adj2 greenness).ti,ab.
16

51 (greenery or greenspace* or greenness).ti,ab.
205

52 (garden$ or park or parks).ti,ab.
8630

53 sports field$.ti,ab.
49

54 wilderness area$.ti,ab.
40

55 public open space$.ti,ab.
41

56 neighbourhood open space$.ti,ab.
0

57 neighborhood open space$.ti,ab.
3

58 (bluespace$ or blue space$).ti,ab.
29

59 bluehealth.ti,ab.
0

60 blue water$.ti,ab.
10

61 blue gym$.ti,ab.
1

62 47 or 48 or 49 or 50 or 51 or 52 or 53 or 54 or 55 or 56 or 57 or 58 or 59 or 60 or 61
26918

63 (air adj2 (quality or pollution)).ti,ab.
1272

64 ((ambient or neighbourhood or neighborhood or outdoor) adj2 air).ti,ab.
280

65 ((traffic or aircraft or industr$ or neighbourhood or neighborhood or outdoor) adj2 noise).ti,ab.
402

66 (noise or (noise adj2 pollution)).ti,ab.
26936

67 63 or 64 or 65 or 66
28225

68 62 or 67
54795

69 46 and 68
15761

70 (health adj promot$ adj2 environment$).ti,ab.
102

71 69 or 70
15856

72 exp Cohort Analysis/
1440

73 ((cohort or longitudinal or follow-up or prospective or retrospective) adj2 stud*).ti,ab.
114282

74 (cohort or follow-up or longitudinal).ti,ab.
262889

75 exp Longitudinal Studies/
16477

76 72 or 73 or 74 or 75
288811

77 71 and 76
998

Science Citation Index via Web of Knowledge

# 30	9,094	#29 AND #26<br>*Indexes=SCI-EXPANDED Timespan=All years*
# 29	2,655,212	#28 OR #27<br>*Indexes=SCI-EXPANDED Timespan=All years*
# 28	364,325	(TS = ((cohort NEAR/2 stud*) OR (longitudin* NEAR/2 stud*) OR (follow-up NEAR/2 stud*))) *AND* **LANGUAGE:** (English)<br>*Indexes=SCI-EXPANDED Timespan=All years*
# 27	2,655,212	((TS= (cohort or longitudin* or follow-up or prospective or retrospective or incidence))) *AND* **LANGUAGE:** (English)<br>*Indexes=SCI-EXPANDED Timespan=All years*
# 26	71,677	(#25 not #23) *AND* **LANGUAGE:** (English)<br>*Indexes=SCI-EXPANDED Timespan=All years*
# 25	74,209	#24 OR #22<br>*Indexes=SCI-EXPANDED Timespan=All years*
# 24	15,983	(TS = ((health NEAR/1 promot* NEAR/1 environment*) or (health promot* environment*))) *AND* **LANGUAGE:** (English)<br>*Indexes=SCI-EXPANDED Timespan=All years*
# 23	2,253,183	(TI=(rat or rats or mouse or mice or bird or birds or cow o r cattle or bovine or sheep or goat* or ovine or horse or equine or pig or pigs or porcine or fish or fishes)) *AND* **LANGUAGE:** (English)<br>*Indexes=SCI-EXPANDED Timespan=All years*

# 22	59,082	#21 AND #5<br>*Indexes=SCI-EXPANDED Timespan=All years*
# 21	339,119	#20 OR #15 OR #11<br>*Indexes=SCI-EXPANDED Timespan=All years*
# 20	162,848	#19 OR #18 OR #17 OR #16<br>*Indexes=SCI-EXPANDED Timespan=All years*
# 19	11,526	(TS = ((traffic or aircraft or industr* or neighbourhood or neighborhood or outdoor or environment*) NEAR/2 noise)) *AND* **LANGUAGE:** (English)<br>*Indexes=SCI-EXPANDED Timespan=All years*
# 18	3,747	(TS = (noise pollution)) *AND* **LANGUAGE:** (English)<br>*Indexes=SCI-EXPANDED Timespan=All years*
# 17	32,621	(TS = ((ambient OR neighbourhood or neighborhood OR outdoor) NEAR/2 air)) *AND* **LANGUAGE:** (English)<br>*Indexes=SCI-EXPANDED Timespan=All years*
# 16	131,233	(TS = (air pollution OR air quality)) *AND* **LANGUAGE:** (English)<br>*Indexes=SCI-EXPANDED Timespan=All years*
# 15	153,909	#14 OR #13 OR #12<br>*Indexes=SCI-EXPANDED Timespan=All years*
# 14	104,846	(TS = (water cover* or blue cover*)) *AND* **LANGUAGE:** (English)<br>*Indexes=SCI-EXPANDED Timespan=All years*
# 13	42,025	(TS = (bluehealth or blue water or blue gym)) *AND* **LANGUAGE:** (English)<br>*Indexes=SCI-EXPANDED Timespan=All years*

# 12	10,091	(TS = (blue space or bluespace)) *AND* **LANGUAGE:** (English)<br>*Indexes=SCI-EXPANDED Timespan=All years*
# 11	27,086	#10 OR #9 OR #8 OR #7 OR #6<br>*Indexes=SCI-EXPANDED Timespan=All years*
# 10	14,251	(TS = (public park OR public parks OR public space OR public open space OR neighbourhood open space OR neighborhood open space)) *AND* **LANGUAGE:** (English)<br>*Indexes=SCI-EXPANDED Timespan=All years*
# 9	324	(TS = ((ambient OR city OR cities OR environment* OR neighbourhood OR neighborhood OR residential OR surrounding OR urban) NEAR/2 greenness)) *AND* **LANGUAGE:** (English)<br>*Indexes=SCI-EXPANDED Timespan=All years*
# 8	6,354	(TS = ((city or cities or environment* or neighbourhood or neighborhood or urban or residential) NEAR/2 greening)) *AND* **LANGUAGE:** (English)<br>*Indexes=SCI-EXPANDED Timespan=All years*
# 7	8,844	(TS = (green NEAR/2 (cover* or environment* or gym* or neighbourhood* or neighborhood* or roadside* or space*))) *AND* **LANGUAGE:** (English)<br>*Indexes=SCI-EXPANDED Timespan=All years*
# 6	4,344	(TS = (greenness OR greenspace OR greenery)) *AND* **LANGUAGE:** (English)<br>*Indexes=SCI-EXPANDED Timespan=All years*
# 5	8,430,658	#4 OR #3 OR #2 OR #1<br>*Indexes=SCI-EXPANDED Timespan=All years*

# 4	1,697,764	(TS= ((health promot* behavio?r*) or diet or (physical activity) or (physical* activ*) or walking or running or exercise or smoking or (alcohol drinking) or (alcohol NEAR/2 consum*))) *AND* **LANGUAGE:** (English)<br><br>*Indexes=SCI-EXPANDED Timespan=All years*
# 3	798,489	(TS = ((physical function*) or (physical functioning) or (quality of life) or ("Quality of Life") or insomnia or sleep or (sleep disrupt*) or frail or frailty)) *AND* **LANGUAGE:** (English)<br><br>*Indexes=SCI-EXPANDED Timespan=All years*
# 2	6,220,283	TS= ((physical health) or cardio-vascular or cardiovascular or (cardio vascular) or myocardial or cancer or respiratory or cardio-respiratory or cardiorespiratory or (cardio respiratory) or diabetes or diabetic or BMI or (Body Mass Index) OR endocrine or musculoskeletal or musculo-skeletal or neoplasms or (digestive system) or digestive or (respiratory tract) or (nervous system) or neurological or eye disease*or (skin and connective tissue) or dermatologic* or (nutritional dis*) or metabolic or endocrine system or immune or HIV o r (human immunodeficiency))<br><br>*Indexes=SCI-EXPANDED Timespan=All years*
# 1	831,083	TS= (depression or depressive or dysthymi* or anxiety or anxiety disorder* or panic disorder* or panic or generali?ed anxiety or obsessive-compulsive disorder* or obsessive-compulsive or obsessive compulsive or ocd or ptsd or posttrauma* or post-traumatic or post traumatic or bipolar or bipolar disorder* or psychotic or (bipolar and (affective or disorder*)) or schizophreni* or psychosis)<br><br>*Indexes=SCI-EXPANDED Timespan=All years*

Scopus

((((TITLE-ABS-KEY (depression OR depressive OR dysthymi* OR anxiety OR (anxiety AND disorder*) OR (panic AND disorder*) OR (panic) OR (generali?ed AND anxiety) OR (obsessive-compulsive AND disorder*) OR (obsessive-compulsive) OR (obsessive AND compulsive) OR ocd OR ptsd OR (posttrauma*) OR post-traumatic OR (post AND traumatic) OR (bipolar) OR (bipolar AND disorder*) OR psychotic OR (bipolar AND (affective OR disorder*)) OR schizophreni* OR psychosis)) OR (TITLE-ABS-KEY ((physical AND health) OR (cardio-vascular) OR (cardiovascular) OR (cardio AND vascular) OR myocardial OR diabetes OR diabetic OR cancer OR respiratory OR (cardio-respiratory) OR cardiorespiratory OR (cardio AND respiratory) OR endocrine OR musculoskeletal OR (musculo-skeletal) OR (neoplasms) OR (digestive AND system) OR digestive OR (respiratory AND tract) OR (nervous AND system) OR neurological OR (eye AND disease*) OR (skin AND connective AND tissue) OR dermatologic* OR (nutrition* AND dis*) OR metabolic OR endocrine OR immune OR hiv OR (human AND immunodeficiency) OR obesity OR bmi OR obese OR (body AND mass AND index))) OR (TITLE-ABS-KEY ((physical AND function*) OR (physical AND functioning) OR (quality AND of AND life) OR ("Quality of Life") OR insomnia OR sleep OR (sleep AND disrupt*) OR frail OR frailty)) OR (TITLE-ABS-KEY ((health AND promot* AND behavio?r*) OR diet OR (physical AND activity) OR (physical* AND activ*) OR walking OR running OR exercise OR smoking OR (alcohol AND drinking) OR (alcohol AND near/2 AND consum*)))) AND ((TITLE-ABS-KEY ((recreational AND park) OR (recreational AND parks))) OR (TITLE-ABS-KEY (garden OR park OR parks OR (public AND park) OR (public AND parks))) OR (TITLE-ABS-KEY (((neighbourhood OR neighborhood OR public) W/2 open AND space))) OR (TITLE-ABS-KEY ((green AND space) OR greenspace)) OR (TITLE-ABS-KEY ((green* W/2 (area OR cover* OR environment OR gym OR neighbourhood OR neighborhood OR roadside OR city OR cities)))) OR (TITLE-ABS-KEY (((area OR neighbourhood OR neighborhood OR roadside OR city OR cities OR urban OR ambient OR residential OR surrounding) W/2 (greening OR greenness)))) OR (TITLE-ABS-KEY (bluespace OR (blue AND space) OR (bluehealth) OR (blue W/2 cover*) OR (blue AND gym) OR (air AND pollution) OR (air AND quality) OR (noise AND pollution) OR ((traffic OR aircraft OR industr* OR neighbourhood OR neighborhood OR outdoor) W/2 noise) OR (noise W/2 pollution))))) OR (TITLE-ABS-KEY ((salutogenic W/2 environment*) OR (health AND promot* W/2 environment*)))) AND (TITLE-ABS-

KEY (cohort OR longitudinal OR follow- AND up OR epidemiol* OR prospective OR retrospective OR incidence)) AND (LIMIT-TO (LANGUAGE , "English"))

GreenFILE via EBSCO

S27
S23 AND S26
(1,026)

S26
S24 OR S25
(13,758)

S25
TI (((cohort or follow-up or longitudinal or prospective or retrospective) N2 study)) OR KW (((cohort or follow-up or longitudinal or prospective or retrospective) N2 study)) OR AB (((cohort or follow-up or longitudinal or prospective or retrospective) N2 study)) (3,453)

S24
TI (cohort or cohorts or follow-up or longitudinal or incidence) OR KW (cohort or cohorts or follow-up or longitudinal or incidence) OR AB (cohort or cohorts or follow-up or longitudinal or incidence)
(13,323)

S23
S21 OR S22
(7,014)

S22
TI (((health promot* or salutogenic) N2 environment*)) OR AB (((health promot* or salutogenic) N2 environment*)) OR KW (((health promot* or salutogenic) N2 environment*))
(14)

S21
S15 AND S20
(7,002)

S20
(S16 OR S17 OR S18 OR S19)
(50,795)

S19
TI (((health promot* behavio?r*) or diet or (physical activity) or (physical* activ*) or walking or running or exercise or smoking or (alcohol drinking) or (alcohol NEAR/2 consum*))) OR AB (((health promot* behavio?r*) or diet or (physical activity) or (physical* activ*) or walking or running or exercise or smoking or (alcohol drinking) or (alcohol NEAR/2 consum*))) OR KW (((health promot* behavio?r*) or diet or (physical activity) or (physical* activ*) or walking or running or exercise o ...
(17,451)

S18
TI (((physical function*) or (physical functioning) or (quality of life) or ("Quality of Life") or insomnia or sleep or (sleep disrupt*) or frail or frailty)) OR AB (((physical function*) or (physical functioning) or (quality of life) or ("Quality of Life") or insomnia or sleep or (sleep disrupt*) or frail or frailty)) OR KW (((physical function*) or (physical functioning) or (quality of life) or ("Quality of Life") or insomnia or sleep or (sleep disrupt*) or frail or frailty))
(1,819)

S17
TI (((physical health) or cardio-vascular or cardiovascular or (cardio vascular) or myocardial or cancer or respiratory or cardio-respiratory or cardiorespiratory or (cardio respiratory) or diabetes or diabetic or BMI or (Body Mass Index) OR endocrine or musculoskeletal or musculo-skeletal or neoplasms or (digestive system) or digestive or (respiratory tract) or (nervous system) or neurological or eye disease*or (skin and connective tissue) or dermatologic* or (nutritional dis*) or metabolic or endocrine system or immune or HIV or (human immunodeficiency))) OR AB (((physical health) or cardio-vascular or cardiovascular or (cardio vascular) or myocardial or cancer or respiratory or cardio-respiratory or cardiorespiratory or (cardio respiratory) or diabetes or diabetic or BMI or (Body Mass Index) OR endocrine or musculoskeletal or musculo-skeletal or neoplasms or (digestive system) or digestive or (respiratory tract) or (nervous system) or neurological or eye disease*or (skin and connective tissue) or dermatologic* or (nutritional dis*) or metabolic or endocrine system or immune or HIV or (human immunodeficiency))) OR KW (((physical health) or cardio-vascular or cardiovascular or (cardio vascular) or myocardial or cancer or respiratory or cardio-respiratory or cardiorespiratory or (cardio respiratory) or diabetes or diabetic or BMI or (Body Mass Index) OR endocrine or musculoskeletal or musculo-skeletal or neoplasms or (digestive system) or digestive or (respiratory tract) or (nervous system) or neurological or eye disease*or (skin and connective tissue) or dermatologic* or (nutritional dis*) or metabolic or endocrine system or immune or HIV or (human immunodeficiency)))
(33,709)

S16
TI ((mental health or depression or depressive or dysthymi* or anxiety or anxiety disorder* or panic disorder* or panic or generali?ed anxiety or obsessive-compulsive disorder* or obsessive-compulsive or obsessive compulsive or ocd or ptsd or posttrauma* or post-traumatic or post traumatic or bipolar or bipolar disorder* or psychotic or (bipolar and (affective or disorder*)) or schizophreni* or psychosis)) OR AB ((mental health or depression or depressive or dysthymi* or anxiety or anxiety disorder* or panic disorder* or panic or generali?ed anxiety or obsessive-compulsive disorder* or obsessive-compulsive or obsessive compulsive or ocd or ptsd or posttrauma* or post-traumatic or post traumatic or bipolar or bipolar disorder* or psychotic or (bipolar and (affective or disorder*)) or schizophreni* or psychosis) greenery) OR KW ((mental health or depression or depressive or dysthymi* or anxiety or anxiety disorder* or panic disorder* or panic or generali?ed anxiety or obsessive-compulsive disorder* or obsessive-compulsive or obsessive compulsive or ocd or ptsd or posttrauma* or post-traumatic or post traumatic or bipolar or bipolar disorder* or psychotic or (bipolar and (affective or disorder*)) or schizophreni* or psychosis))
(926)

S15
S1 OR S2 OR S3 OR S4 OR S5 OR S6 OR S7 OR S8 OR S9 OR S10 OR S11 OR S12 OR S13 OR S14
(88,879)

S14
TI (coast* or water cover* or blue cover*) OR AB (coast* or water cover* or blue cover*) OR KW (coast* or water cover* or blue cover*)

S13
TI (((traffic or aircraft or industr* or neighbourhood or outdoor) N2 noise)) OR AB (((traffic or aircraft or industr* or neighbourhood or outdoor) N2 noise)) OR KW (((traffic or aircraft or industr* or neighbourhood or outdoor) N2 noise))

S12
TI (noise or noise pollution) OR AB (noise or noise pollution) OR KW (noise or noise pollution)

S11
TI (((ambient OR neighbourhood OR outdoor) N2 air)) OR AB (((ambient OR neighbourhood OR outdoor) N2 air)) OR KW (((ambient OR neighbourhood OR outdoor) N2 air))

S10
TI (air pollution OR air quality) OR AB (air pollution OR air quality) OR KW (air pollution OR air quality)

S9
TI (blue space or bluespace) OR AB (blue space or bluespace) OR KW (blue space or bluespace)

S8
TI (bluehealth or blue water or blue gym) OR AB (bluehealth or blue water or blue gym) OR KW (bluehealth or blue water or blue gym)

S7
TI ((neighbourhood or neighborhood) N2 open space) OR AB ((neighbourhood or neighborhood) N2 open space) OR KW ((neighbourhood or neighborhood) N2 open space)

S6
TI (public N2 (open space or park or parks or space)) OR AB (public N2 (open space or park or parks or space)) OR KW (public N2 (open space or park or parks or space))

S5
TI (garden or park or parks or sports field) OR AB (garden or park or parks or sports field) OR KW (garden or park or parks or sports field)

S4
TI (((ambient or city or cities or environment* or neighbourhood or neighborhood or residential or surrounding or urban) N2 greenness)) OR AB (((ambient or city or cities or environment* or neighbourhood or neighborhood or residential or surrounding or urban) N2 greenness)) OR KW (((ambient or city or cities or environment* or neighbourhood or neighborhood or residential or surrounding or urban) N2 greenness))

S3
TI (((city or cities or environment* or neighbourhood or neighborhood or urban) N2 greening)) OR AB (((city or cities or environment* or neighbourhood or neighborhood or urban) N2 greening)) OR KW (((city or cities or environment* or neighbourhood or neighborhood or urban) N2 greening))

S2

TI ((green N2 (area* or cover or environment* or gym* or neighbourhood* or neighborhood* or roadside* or space*)).) OR AB ((green N2 (area* or cover or environment* or gym* or neighbourhood* or neighborhood* or roadside* or space*)).) OR KW ((green N2 (area* or cover or environment* or gym* or neighbourhood* or neighborhood* or roadside* or space*)))

S1

TI (green space OR greenspace OR greenery) OR AB (green space OR greenspace OR greenery) OR KW (green space OR greenspace OR greenery)
