# Supplementary material for: Relationship between Green and Blue Spaces with Mental and Physical Health: A Systematic Review of Longitudinal Observational Studies
Source: Int J Environ Res Public Health. 2021 Aug 26;18(17):9010. doi: 10.3390/ijerph18179010 (PMC8431638; doi:10.3390/ijerph18179010)
Supplement: Supplementary file 1 [file ijerph-18-09010-s001.zip › ijerph-1307799-supplementary/Supplementary material S2.pdf]

Supplementary Material 2: Data Extraction Form Adapted from Cochrane (Higgin et al., 2019) [47]

Study No.

Date form completed (dd/mm/yyyy)
Name/ID of person extracting data
Report title
(title of paper/ abstract/ report that data are extracted from)
Report ID
Reference details
Reference details
Report author contact details
Publication type

Study funding source
(including role of funders)
Possible conflicts of interest

Notes:

Eligibility

Is the Study Longitudinal and Observational?
Population
Exposure
(type/ characteristics)

Health Outcome

Study Methods

Population Description

Setting/ Source

Cohort name/ data source description

Prospective or Retrospective Study?

Methods of Participant Recruitment

Aims/ Objectives

Duration of Follow -up

Participants

Sample Size

Total Sample Size

Age Group

Sex

Ethnicity

Socio-economic Status

Other Characteristics

Exposures

Type
(e.g green, blue space)

Definition

Characteristics Description
(e.g access, distance, proportion)

Exposure Metrics Used

Method of Measuring Exposure
(e.g Satellite imaging, interviews)

Method of Exposure Assignment
(e.g GIS, census data)

Unit of Analysis
(e.g. individual, area)

Additional Exposures
(those not studied in the review)

Comparator Group
(e.g no exposure; exposure to different environmental variable)

Primary Outcomes

Outcome/s Type
(e.g. behaviour, mental/physical health condition)

Outcome Definition

Measurement Tools
(e.g. self-reported instrument; clinician assessment)

Outcome Variable Type
(e.g dichotomous, continuous variable)

Stats

Additional Outcomes

Outcome/s Type
(e.g. behaviour, mental/physical health condition)

Outcome Definition

Measurement Tools
(e.g. validated, self-reported instrument; clinician assessment)

Time Points Recorded

Effect Estimate of Outcome/s

Results

Outcome/s

Results
(effect estimate & variance)

Type of Effect Estimate (e.g Odds Ratio, Incidence ratio, Beta, mean)

Unit of Analysis
(e.g individual, group)

Confounders

Statistical Methods

Appropriateness of Statistical Methods Used

Subgroup Analyses

Mediators
(if any)

Loss to Follow-Up Number and Reasons

Handling of Missing Data

Discussion

Main Discussion Points

Other Notes

Conclusion and Limitations

Limitations

Strengths

Strategies to Overcome Limitations

Conclusions

Notes

Other Information

Does the study directly address review question?

References to other relevant studies
