# Supplementary material for: Relationship between Green and Blue Spaces with Mental and Physical Health: A Systematic Review of Longitudinal Observational Studies
Source: Int J Environ Res Public Health. 2021 Aug 26;18(17):9010. doi: 10.3390/ijerph18179010 (PMC8431638; doi:10.3390/ijerph18179010)
Supplement: Supplementary file 1 [file ijerph-18-09010-s001.zip › ijerph-1307799-supplementary/Supplementary material S3.pdf]

Newcastle-Ottawa Quality Assessment Form for Cohort Studies

Note: A study can be given a maximum of one star for each numbered item within the Selection and Outcome categories. A maximum of two stars can be given for Comparability.

Selection

1) Representativeness of the exposed cohort
    a) Truly representative (one star)
    b) Somewhat representative (one star)
    c) Selected group
    d) No description of the derivation of the cohort
2) Selection of the non-exposed cohort
    a) Drawn from the same community as the exposed cohort (one star)
    b) Drawn from a different source
    c) No description of the derivation of the non exposed cohort
3) Ascertainment of exposure
    a) Secure record (e.g., surgical record) (one star)
    b) Structured interview (one star)
    c) Written self report
    d) No description
    e) Other
4) Demonstration that outcome of interest was not present at start of study
    a) Yes (one star)
    b) No

Comparability

1) Comparability of cohorts on the basis of the design or analysis controlled for confounders

a) The study controls for age, sex and marital status (one star)

b) Study controls for other factors (list) education and/or income and/or deprivation (one star)

c) Cohorts are not comparable on the basis of the design or analysis controlled for confounders

Outcome

1) Assessment of outcome

   a) Independent blind assessment (one star)

   b) Record linkage (one star)

   c) Self report

   d) No description

   e) Other

2) Was follow-up long enough for outcomes to occur

   a) Yes (one star)

   b) No

   Indicate the median duration of follow-up and a brief rationale for the assessment above: median duration of follow-up was decided on study individual basis considering the outcome of interest (eg type of mental health condition, NCD, health behaviour)

3) Adequacy of follow-up of cohorts

   a) Complete follow up- all subject accounted for (one star)

   b) Subjects lost to follow up unlikely to introduce bias- number lost less than or equal to 20% or description of those lost suggested no different from those followed. (one star)

   c) Follow up rate less than 80% and no description of those lost

   d) No statement

Thresholds for converting the Newcastle-Ottawa scales to AHRQ standards (good, fair, and poor):

Good quality: 3 or 4 stars in selection domain AND 1 or 2 stars in comparability domain AND 2 or 3 stars in outcome/exposure domain

Fair quality: 2 stars in selection domain AND 1 or 2 stars in comparability domain AND 2 or 3 stars in outcome/exposure domain

Poor quality: 0 or 1 star in selection domain OR 0 stars in comparability domain OR 0 or 1 stars in outcome/exposure domain
