# Supplementary material for: Relationship between Green and Blue Spaces with Mental and Physical Health: A Systematic Review of Longitudinal Observational Studies
Source: Int J Environ Res Public Health. 2021 Aug 26;18(17):9010. doi: 10.3390/ijerph18179010 (PMC8431638; doi:10.3390/ijerph18179010)
Supplement: Supplementary file 1 [file ijerph-18-09010-s001.zip › ijerph-1307799-supplementary/Supplementary material S4.pdf]

Supplementary Material 4: Table of Excluded Studies During Full-text Screening

Study No.	Study Title	Reason for exclusion
1.	Enjoying gardening produces desirable cardiovascular prognosis by maintaining favorable coronary endothelial function	conference paper
2.	Urban green space, tree canopy, and prevention of heart disease, hypertension, and diabetes: a longitudinal study	conference paper
3.	The relationship of neighborhood environment to sleep apnea: The multi-ethnic study of atherosclerosis	conference paper
4.	Obesity and built environment: Does the association hold longitudinally?	conference paper
5.	Obesogenic microenvronment, sleep patterns and metabolic profiles in an overweight and obese pregnant population	conference paper
6.	The obesogenic environment in an overweight and obese pregnant population	conference paper
7.	Associations of community and environmental factors with 6-month transition states of chronic rhinosinusitis	conference paper
8.	Neighborhood obesogenic environment and the risk of prostate cancer: The Multiethnic Cohort	conference paper
9.	Neighborhood green qualities, social capital and safety in relation to well-being and a healthy lifestyle: A longitudinal study	conference paper
10.	Association between the built environment and heart failure progression in an urban retrospective cohort study	conference paper
11.	How might the built environment influence obesity? assessment of mediation by physical activity and sedentary behavior	conference paper?
12.	Does area of residence influence weight loss following a diagnosis of type 2 diabetes? Fixed effects longitudinal analysis of 54,707 middle-to-older aged Australians	conference paper/ communication

13.	Access to green space, physical activity and mental health: a twin study	cross-sectional
14.	Coastal blue space and depression in older adults	cross-sectional
15.	Associations between time spent in green areas and physical activity among late middle-aged adults	cross-sectional
16.	Availability of recreational resources and physical activity in adults	cross-sectional
17.	Individual-level exposure to disaster, neighborhood environmental characteristics, and their independent and combined associations with depressive symptoms in women	data from experimental
18.	A Longitudinal Analysis of the Influence of the Neighborhood Environment on Recreational Walking within the Neighborhood: Results from RESIDE	data from experimental study
19.	Residential greenness and increased physical activity in patients after coronary artery bypass graft surgery	data from experimental study
20.	Why neighborhood park proximity is not associated with total physical activity	data from experimental study
21.	Environmental facilitators for outdoor walking and development of walking difficulty in community-dwelling older adults	data from experimental study
22.	The impact of greenery on physical activity and mental health of adolescent and adult residents of deprived neighborhoods: A longitudinal study	data from experimental study
23.	Etiology of lung cancer and environmental risk factors in Xuanwei, China	dissertation
24.	Residential greenspace and lung function up to 24¬†years of age: The ALSPAC birth cohort	duplicate
25.	Green space-a novel risk decreasing mechanism for schizophrenia?	duplicate

26.	Developing a data-driven spatial approach to assessment of neighbourhood influences on the spatial distribution of myocardial infarction	exposure: exclude
27.	The protective effect of neighborhood composition on increasing frailty among older Mexican Americans: a barrio advantage?	exposure: no GS
28.	Neighborhood Health-Promoting Resources and Obesity Risk (the Multi-Ethnic Study of Atherosclerosis)	exposure: no GS
29.	Neighborhood Physical Environment and Changes in Body Mass Index: Results From the Multi-Ethnic Study of Atherosclerosis	exposure: no GS
30.	Local descriptive norms for overweight/obesity and physical inactivity, features of the built environment, and 10-year change in glycosylated haemoglobin in an Australian population-based biomedical cohort	exposure: no GS
31.	Longitudinal Associations Between Neighborhood Physical and Social Environments and Incident Type 2 Diabetes Mellitus: The Multi-Ethnic Study of Atherosclerosis (MESA)	exposure: no GS
32.	Associations between the perceived environment and physical activity among adults aged 55-65 years: does urban-rural area of residence matter?	exposure: no GS
33.	Associations between neighbourhood characteristics and depression: a twin study	exposure: no GS
34.	The role of personal values, urban form, and auto availability in the analysis of walking for transportation	exposure: no GS
35.	Community-based study of stroke incidence in the Valley of Aosta, Italy. CARe-cerebrovascular Aosta Registry: years 2004-2005	exposure: no GS
36.	Breast cancer survivors involved in vigorous team physical activity: Psychosocial correlates of maintenance participation	exposure: no GS
37.	Determinants of change in airway reactivity over 11 years in the SAPALDIA population study	exposure: no GS
38.	Prevalence of sarcoidosis in Switzerland is associated with environmental factors	exposure: no GS
39.	Estimated change in physical activity level (PAL) and prediction of 5-year weight change in men: the Aerobics Center Longitudinal Study	exposure: no GS
40.	Age-related socio-economic and geographic disparities in breast cancer stage at diagnosis: a population-based study	exposure: no GS

41.	The interaction of physical, psychological, socioeconomic and sociodemographic variables on the Body Mass Index (MINDEX) of the community-dwelling elderly	exposure: no GS
42.	Physical Activity through Sustainable Transport Approaches (PASTA): protocol for a multi-centre, longitudinal study	exposure: no GS
43.	Environmental factors and social adjustment as predictors of a first psychosis in subjects at ultra high risk	exposure: no GS
44.	Educational level and decreases in leisure time physical activity: predictors from the longitudinal GLOBE study	exposure: no GS
45.	Register analysis of measures of urbanization and cancer incidence in Sweden	exposure: no GS
46.	A pilot study evaluating genetic and environmental factors for postpartum depression	exposure: no GS
47.	Epidemiology of wilderness search and rescue in New Hampshire, 1999-2001	exposure: no GS
48.	Does area of residence affect body size and shape?	exposure: no GS
49.	Degree of urbanization and mammographic density in Dutch breast cancer screening participants: results from the EPIC-NL cohort	exposure: no GS
50.	Barriers to stroke thrombolysis in a geographically defined population	exposure: no GS
51.	Residential area characteristics and disabilities among Dutch community-dwelling older adults	exposure: no GS
52.	Changes in neighborhood characteristics and depression among sexual minority young adults	exposure: no GS
53.	Association Between Active Commuting and Incident Cardiovascular Diseases in Chinese: A Prospective Cohort Study	exposure: no GS
54.	Gender Differences in Geriatric Depressive Symptoms in Rural China: The Role of Physical Housing Environments and Living Arrangements	exposure: no GS
55.	Spatial heterogeneity of the relationships between environmental characteristics and active commuting: towards a locally varying social ecological model	exposure: no GS

56.	URBAN RURAL VARIATION IN CANCER INCIDENCE IN DENMARK 1943-1987	exposure: no GS
57.	Living in a Well-Serviced Urban Area Is Associated With Maintenance of Frequent Walking Among Seniors in the VoisiNuAge Study	exposure: no GS
58.	Neighborhood social and physical environments and type 2 diabetes mellitus in African Americans: The Jackson Heart Study	exposure: no GS
59.	Residential environments and smoking behaviour patterns among young adults: A prospective study using data from the Interdisciplinary Study of Inequalities in Smoking cohort	exposure: no GS
60.	The Impact of Neighborhood Environment, Social Support, and Avoidance Coping on Depressive Symptoms of Pregnant African-American Women	exposure: no GS
61.	Influence of age, sex, and place of residence on clinical expression of giant cell arteritis in northwest Spain	exposure: no GS
62.	A critical assessment of geographic clusters of breast and lung cancer incidences among residents living near the Tittabawassee and Saginaw Rivers, Michigan, USA	exposure: no GS
63.	Neighbourhood environment and stroke: a follow-up study in Sweden	exposure: no GS
64.	Productive Activities and Psychological Well-Being Among Older Adults	exposure: no GS
65.	Residence of incident cohort of psychotic patients after 13 years of follow up	exposure: no GS
66.	Neighborhood Social Context and Kidney Function Over Time: The multi-Ethnic Study of Atherosclerosis	exposure: no GS
67.	Built environment change and change in BMI and waist circumference: Multi-ethnic Study of Atherosclerosis	exposure: no GS
68.	Changes in the built environment and changes in the amount of walking over time: longitudinal results from the multi-ethnic study of atherosclerosis	exposure: no GS
69.	Geographic Variations and Time Trends in Cancer Treatments in Taiwan	exposure: no GS
70.	Space-Time Statistical Insights about Geographic Variation in Lung Cancer Incidence Rates: Florida, USA, 2000-2011	exposure: no GS
71.	Impact of occupational environmental stressors on blood pressure changes and on incident cases of hypertension: a 5-year follow-up from the VISAT study	exposure: no GS

72.	Changes in the food environment over time: examining 40 years of data in the Framingham Heart Study	exposure: no GS
73.	The Walking Renaissance: A Longitudinal Analysis of Walking Travel in the Greater Los Angeles Area, USA	exposure: no GS
74.	Toward a multidimensional understanding of residential neighborhood: a latent profile analysis of Los Angeles neighborhoods and longitudinal adult excess weight	exposure: no GS
75.	Residential relocation trajectories and neighborhood density, mixed land use and access networks as predictors of walking and bicycling in the Northern Finland Birth Cohort 1966	exposure: no GS
76.	Evidence that the urban environment specifically impacts on the psychotic but not the affective dimension of bipolar disorder	exposure: no GS
77.	Schizophrenia and the city: A review of literature and prospective study of psychosis and urbanicity in Ireland	exposure: no GS
78.	A longitudinal twin study of 1-year prevalence of major depression in women	exposure: no GS
79.	Geographical variation and incidence of inflammatory bowel disease among US women	exposure: no GS
80.	Objectively Measured Neighborhood Walkability and Change in Physical Activity in Older Japanese Adults: A Five-Year Cohort Study	exposure: no GS
81.	Physical activity behavior change in middle-aged and older women: the role of barriers and of environmental characteristics	exposure: no GS
82.	Physical Activity in the Summer Heat: How Hot Weather Moderates the Relationship Between Built Environment Features and Outdoor Physical Activity of Adults	exposure: no GS
83.	The built environment and physical activity levels: the Harvard Alumni Health Study	exposure: no GS
84.	Safely active mobility for urban baby boomers: The role of neighborhood design	exposure: no GS
85.	Spatial analysis of gastric cancer morbidity in regions of rapid urbanization: a case study in Xiamen, China	exposure: no GS
86.	Neighborhood environment, social mobility, and health: A longitudinal examination of individual and neighborhood factors	exposure: no GS

87.	Supportive neighbourhood built characteristics and dog-walking in Canadian adults	exposure: no GS
88.	Road, rail, and air transportation noise in residential and workplace neighborhoods and blood pressure (RECORD Study)	exposure: no GS
89.	Predictors of initiating and maintaining active commuting to work using transport and public health perspectives in Australia	exposure: no GS
90.	The urban built environment and associations with women's psychosocial health	exposure: no GS
91.	Built environment and lower extremity physical performance: prospective findings from the study of osteoporotic fractures in women	exposure: no GS
92.	Early alcohol use, rural residence, and adult employment	exposure: no GS
93.	Accumulated exposure to rural areas of residence over the life course is associated with overweight and obesity in adulthood: a 25-year prospective cohort study	exposure: no GS
94.	Physical Limitations, Walkability, Perceived Environmental Facilitators and Physical Activity of Older Adults in Finland	exposure: no GS
95.	Longitudinal associations between neighborhood recreational facilities and change in recreational physical activity in the multi-ethnic study of atherosclerosis, 2000-2007	exposure: no GS
96.	Unmet Physical Activity Need in Old Age	exposure: no GS
97.	Fear of moving outdoors and development of outdoor walking difficulty in older people	exposure: no GS
98.	Perceived environmental barriers to outdoor mobility and changes in sense of autonomy in participation outdoors among older people: a prospective two-year cohort study	exposure: no GS
99.	Mobility Modification Alleviates Environmental Influence on Incident Mobility Difficulty among Community-Dwelling Older People: A Two-Year Follow-Up Study	exposure: no GS
100.	The role of environmental factors for the onset of restricted mobility outside the home among older adults with osteoarthritis: a prospective cohort study	exposure: no GS
101.	Assessing the Potential of Land Use Modification to Mitigate Ambient NO2 and Its Consequences for Respiratory Health	exposure: no GS
102.	Individual and Combined Effects of Environmental Risk Factors for Esophageal Cancer Based on Results From the Golestan Cohort Study	exposure: no GS

103.	Associations between recreational walking and attractiveness, size, and proximity of neighborhood open spaces	exposure: no GS
104.	Residential area and physical activity: A multi-level study of 68,000 adults in Stockholm County	exposure: no GS
105.	Associations between perceived neighbourhood problems and quality of life in older adults with and without osteoarthritis: Results from the Hertfordshire Cohort Study	exposure: no GS
106.	Associations between neighborhood perceptions and mental well-being among older adults	exposure: no GS
107.	Five-year predictors of physical activity decline among adults in low-income communities: a prospective study	exposure: no GS
108.	Perceived and objective characteristics of the neighborhood environment are associated with accelerometer-measured sedentary time and physical activity, the CARDIA Study	exposure: no GS
109.	Parks and health: differences in constraints and negotiation strategies for park-based leisure time physical activity by stage of change	exposure: no GS
110.	How the built environment affects change in older people's physical activity: A mixed- methods approach using longitudinal health survey data in urban China	exposure: no GS
111.	Neighborhood environment and loss of physical function in older adults: Evidence from the Alameda County Study	exposure: no GS
112.	Disablement in Context: Neighborhood Characteristics and Their Association With Frailty Onset Among Older Adults	exposure: no GS
113.	Lung cancer, smoking, and environment: A cohort study of the Danish population: BMJ BMJ	exposure: no GS
114.	Investigating the association of neighborhood walkability with risk factors for cardiovascular disease among recently hospitalized patients with systolic heart failure	exposure: no GS
115.	Individual, social, and physical environmental factors related to changes in walking and cycling for transport among older adults: A longitudinal study	exposure: no GS
116.	[The environment, life style and pregnancy outcome]	language
117.	[Lung cancer in the province of Trieste]	language

118.	Environmental factors in chronic delirium	language
119.	Residential green space quantity and quality and symptoms of psychological distress: a 15-year longitudinal study of 3897 women in postpartum	outcome not included
120.	Association between urban green space and the risk of cardiovascular disease: A longitudinal study in seven Korean metropolitan areas	outcome: mortality
121.	The association between neighborhood greenness and incidence of lethal prostate cancer: A prospective cohort study	outcome: mortality
122.	Neighborhood influences on recreational physical activity and survival after breast cancer	outcome: mortality
123.	Does sleep grow on trees? A longitudinal study to investigate potential prevention of insufficient sleep with different types of urban green space	outcome: no validated instrument used
124.	Have walking and bicycling increased in the US? A 13-year longitudinal analysis of traffic counts from 13 metropolitan areas	outcome: no validated instrument used
125.	Initiating and maintaining recreational walking: A longitudinal study on the influence of neighborhood green space	outcome: no validated instrument used
126.	Benefits of walking and solo experiences in UK wild places	outcome: no validated instrument used
127.	What accounts for 'England's green and pleasant land'? A panel data analysis of mental health and land cover types in rural England	outcome: not in ICD
128.	Green qualities in the neighbourhood and mental health - results from a longitudinal cohort study in Southern Sweden	outcome: not in ICD
129.	Contributions of Multiple Built Environment Features to 10-Year Change in Body Mass Index and Waist Circumference in a South Australian Middle-Aged Cohort	outcome: not in ICD
130.	Changes in perceptions of urban green space are related to changes in psychological well-being: Cross-sectional and longitudinal study of mid-aged urban residents	outcome: not in ICD
131.	Inequality, green spaces, and pregnant women: Roles of ethnicity and individual and neighbourhood socioeconomic status	outcome: not in ICD
132.	Residential green and blue space associated with better mental health: a pilot follow-up study in university students	outcome: not in ICD
133.	Residential surrounding green, air pollution, traffic noise and self-perceived general health	outcome: not in ICD

134.	Association between neighborhood greenspace and fasting plasma glucose from a large cohort study in Taiwan	outcome: not in ICD
135.	Body mass index, safety hazards, and neighborhood attractiveness	outcome: not in ICD
136.	Effect of changes in green spaces on mental health in older adults: a fixed effects analysis	outcome: not in ICD
137.	Effects of changing exposure to neighbourhood greenness on general and mental health: A longitudinal study	outcome: not in ICD
138.	Change in Neighborhood Characteristics and Change in Coronary Artery Calcium: A Longitudinal Investigation in the MESA (Multi-Ethnic Study of Atherosclerosis) Cohort	outcome: not in ICD
139.	Can green space quantity and quality help prevent postpartum weight gain? A longitudinal study	outcome: not in ICD
140.	Blood pressure in young adulthood and residential greenness in the early-life environment of twins	outcome: not in ICD
141.	Long-Term Greenspace Exposure and Progression of Arterial Stiffness: The Whitehall II Cohort Study	outcome: not in ICD
142.	Residential greenspace and lung function up to 24 years of age: The ALSPAC birth cohort	outcome: not in ICD
143.	Residential greenness and lung function in a prospective cohort of European adults: The ECRHS study	outcome: not in ICD
144.	Land-Use Change and Cardiometabolic Risk Factors in an Urbanizing Area of South India: A Population-Based Cohort Study	outcome: not in ICD
145.	Would increasing access to recreational places promote healthier weights and a healthier nation?	outcome: not in ICD
146.	Neighbourhood physical activity environments and adiposity in children and mothers: a three-year longitudinal study	outcome: not in ICD
147.	Coastal proximity, health and well-being: Results from a longitudinal panel survey	outcome: not in ICD
148.	Associations of types of green space across the life-course with blood pressure and body mass index	**outcome: not in ICD but good for thesis**
149.	The association between green space and mental health varies across the lifecourse. A longitudinal study	outcome: not in inclusion criteria

150.	Swimming in the USA: beachgoer characteristics and health outcomes at US marine and freshwater beaches	outcome: not in inclusion criteria
151.	Geographic variation in the treatment of non-ST-segment myocardial infarction in the English National Health Service: a cohort study	outcome: not in inclusion criteria
152.	Association between access to health-promoting facilities and participation in cardiovascular disease (CVD) risk screening among populations with low socioeconomic status (SES) in Singapore	outcome: screening behaviour
153.	Exploring the Relevance of Green Space and Epidemic Diseases Based on Panel Data in China from 2007 to 2016	outcome: infectious disease
154.	Neighbourhood-level air pollution and greenspace and inflammation in adults	outcome: inflammation
155.	Contributions of environment, comorbidity, and stage of dementia to the onset of walking and eating disability in long-term care residents	outcome: dementia
156.	The role of urban environment, social and health determinants in the tracking of leisure-time physical activity throughout adolescence	population, children
157.	Urban form relationships with walk trip frequency and distance among youth	population, children
158.	Context Matters: Adolescent Neighborhood and School Influences on Young Adult Body Mass Index	population, children
159.	Childhood exposure to green space-a novel risk-decreasing mechanism for schizophrenia?	population: children
160.	Residential green space in childhood is associated with lower risk of psychiatric disorders from adolescence into adulthood	population: children
161.	Childhood exposure to green space - A novel risk-decreasing mechanism for schizophrenia?	population: children
162.	How do they do it: working women meeting physical activity recommendations	study type: case-control
163.	HABITAT: A longitudinal multilevel study of physical activity change in mid-aged adults	study type: cross-sectional
164.	International Mind, Activities and Urban Places (iMAP) study: methods of a cohort study on environmental and lifestyle influences on brain and cognitive health	study type: cross-sectional
165.	Mental health in the slums of Dhaka - a geoepidemiological study	study type: cross-sectional

166.	Association between community greenness and obesity in urban-dwelling Chinese adults	study type: cross-sectional
167.	GPS-Based Exposure to Greenness and Walkability and Accelerometry-Based Physical Activity	study type: cross-sectional
168.	Residential Air Pollution, Road Traffic, Greenness and Maternal Hypertension: Results from GINIplus and LISAplus	study type: cross-sectional
169.	Cardiac arrest while exercising on mountains in national or provincial parks: A national observational study from 2012 to 2015	study type: cross-sectional
170.	Associations of perceived neighborhood environment on health status outcomes in persons with arthritis	study type: cross-sectional
171.	Relationships between neighborhoods, physical activity, and obesity: A multilevel analysis of a large Canadian city	study type: cross-sectional
172.	Activity space environment and dietary and physical activity behaviors: a pilot study	study type: cross-sectional
173.	Green space associations with mental health and cognitive function: Results from the Quebec CARTaGENE cohort	study type: cross-sectional
174.	Multiple dimensions of residential environments, neighborhood experiences, and jogging behavior in the record study	study type: cross-sectional
175.	Effect of Social Factors and the Natural Environment on the Etiology and Pathogenesis of Diabetes Mellitus	study type: discussion
176.	Residential moves, neighbourhood walkability, and physical activity: a longitudinal pilot study in Ontario Canada	study type: experimental
177.	The aftermath of public housing relocations: relationships between changes in local socioeconomic conditions and depressive symptoms in a cohort of adult relocaters	study type: experimental
178.	Convergence of prevalence rates of diabetes and cardiometabolic risk factors in middle and low income groups in urban India: 10-year follow-up of the Chennai Urban Population Study	study type: experimental
179.	Rural--urban differences in health and health behaviour: A baseline description of a community health-promotion programme for the elderly	study type: experimental
180.	Causal evaluation of urban greenway retrofit: A longitudinal study on physical activity and sedentary behavior	study type: experimental
181.	The causal influence of neighborhood design on physical activity within the neighborhood: evidence from Northern California	study type: experimental
182.	Changes in neighborhood walking are related to changes in perceptions of environmental attributes	study type: experimental

183.	Physical and Mental Health Impacts of Household Gardens in an Urban Slum in Lima, Peru	study type: experimental
184.	Walking Green: Developing an Evidence Base for Nature Prescriptions	study type: experimental
185.	A microenvironment approach to reducing sedentary time and increasing physical activity of children and adults at a playground	study type: experimental
186.	Benefits for Older People Engaged in Environmental Volunteering and Socializing Activities in City Parks: Preliminary Results of a Program in Italy	study type: experimental
187.	A longitudinal examination of improved access on park use and physical activity in a low-income and majority African American neighborhood park	study type: experimental
188.	Environmental stressors and cardio-metabolic disease: part I-epidemiologic evidence supporting a role for noise and air pollution and effects of mitigation strategies	study type: literature review
189.	Obesity, diet quality, physical activity, and the built environment: the need for behavioral pathways	study: cross-sectional
