# Supplementary material for: Relationship between Green and Blue Spaces with Mental and Physical Health: A Systematic Review of Longitudinal Observational Studies
Source: Int J Environ Res Public Health. 2021 Aug 26;18(17):9010. doi: 10.3390/ijerph18179010 (PMC8431638; doi:10.3390/ijerph18179010)
Supplement: Supplementary file 1 [file ijerph-18-09010-s001.zip › ijerph-1307799-supplementary/Supplementary material S5.pdf]

Supplementary Material 5: Table of studies' NOS rating

Study	Selection				Comparability (confounding)		Outcome			
Column1	Representativeness of the exposed	Selection of the non-exposed	Ascertainment of	Demonstration outcome of interest not present at start of study	Main confounders	Additional Confounders	Outcome assessment	Length of Follow-up	Adequacy of follow-up	Quality
Lee et al., 2017	-	*	*	-	*	*	*	*	-	Fair
De keijzer et al., 2019b	-	*	*	-	*	*	*	*	*	Fair
Michael et al., 2010	-	*	*	-	*	*	-	*	*	Fair
Dalton et al., 2016 b	-	*	*	-	*	*	-	*	*	Fair
Hobbs et al., 2019	-	*	*	-	*	*	-	*	*	Fair
Lin et al., 2020	-	*	*	-	*	*	-	*	*	Fair
Chang et al., 2019	*	*	*	*	*	*	*	*	-	Good
Gariepy et al., 2015	*	*	*	-	*	*	-	*	*	Good
Tomita et al., 2017	*	*	*	*	*	*	-	*	*	Good
Paquet et al., 2014	*	*	*	-	*	*	*	*	-	Good
De keijzer et al., 2019a	-	*	*	*	*	*	*	*	-	Good
Dalton and jones, 2020	-	*	*	*	*	*	*	*	-	Good
Paul et al., 2020	-	*	*	*	*	*	*	*	-	Good
Orioli et al., 2019	*	*	*	*	*	*	*	*	-	Good

Tamosiunas et al., 2014	*	*	*	*	*	*	-	*	*	Good
Pun et al., 2018	*	*	*	-	*	*	-	*	*	Good
Clark et al., 2017	*	*	*	*	*	*	*	*	*	Good
Datzman et al.,	-	*	*	*	*	*	*	*	-	Good
Conroy et al., 2017	*	*	*	*	*	*	*	*	-	Good
Yu et al., 2018	*	*	*	-	*	*	*	*	*	Good
Liao et al., 2019	-	*	*	*	*	*	*	*	*	Good
Persson et al., 2018	*	*	*	-	*	*	*	*	*	Good
Yuchi et al., 2020	*	*	*	*	*	*	*	*	-	Good
Zhu et al., 2020	*	*	*	-	*	*	-	*	*	Good
Fernandez-nino et al., 2019	-	*	*	*	*	*	-	*	*	Good
Gariepy et al., 2015	-	*	*	*	*	*	-	*	*	Good
Haraldsdottir et al., 2017	*	*	-	*	*	*	*	*	*	Good
Dalton et al., 2016a	-	*	*	*	*	*	-	*	*	Good
Melis et al.,2015	*	*	*	*	*	*	*	*	*	Good
Renzi et al., 2018	*	*	*	*	*	*	*	*	*	Good
Banay ey al., 2019	*	*	*	*	*	*	-	*	-	Poor
Meyer et al., 2015	-	*	*	-	*	*	-	*	-	Poor
Chong et al., 2019	*	*	*	-	*	*	-	*	-	Poor
Josey and moore., 2018	-	*	*	-	*	*	-	*	-	Poor
Yang et al., 2017	-	*	*	-	*	-	-	*	-	Poor
Cleland et al., 2009	*	*	-	-	*	*	-	*	-	Poor

Halonen et al., 2014	*	*	*	-	*	*	-	*	-	Poor
Picavet et al., 2016	-	*	*	-	*	*	-	*	-	Poor
Hogendorf et al., 2020	-	*	*	-	*	*	-	*	-	Poor
Coogan et al., 2009	*	*	*	-	*	*	-	*	-	Poor
Sugiyama et al., 2015	*	*	-	-	*	*	-	*	-	Poor
Astell-burt and feng, 2020	*	*	*	-	*	*	-	*	-	Poor
Astell-burt and feng, 2019	*	*	*	*	*	*	-	*	-	Poor
Faerstein et al., 2018	-	*	*	-	*	*	-	*	-	Poor

Thresholds for converting the Newcastle-Ottawa scales to AHRQ standards (good, fair, and poor):

Good quality: 3 or 4 stars in selection domain AND 1 or 2 stars in comparability domain AND 2 or 3 stars in outcome/exposure domain

Fair quality: 2 stars in selection domain AND 1 or 2 stars in comparability domain AND 2 or 3 stars in outcome/exposure domain

Poor quality: 0 or 1 star in selection domain OR 0 stars in comparability domain OR 0 or 1 stars in outcome/exposure domain
