# Supplementary material for: Relationship between Green and Blue Spaces with Mental and Physical Health: A Systematic Review of Longitudinal Observational Studies
Source: Int J Environ Res Public Health. 2021 Aug 26;18(17):9010. doi: 10.3390/ijerph18179010 (PMC8431638; doi:10.3390/ijerph18179010)
Supplement: Supplementary file 1 [file ijerph-18-09010-s001.zip › ijerph-1307799-supplementary/Supplementary Material S6.pdf]

Supplementary Material 6: Summary of studies' sensitivity analyses

Reference	Population Description	Duration of Follow-up	Exposure Description	Outcome Description	Outcome Measure	Main results	Effect Estimate	Confounders	Sensitivity analyses	Sensitivity Analysis results
Meyer et al., 2015	men and women; ≥ 18-30 years; black and white; (USA)	13 years	Number of parks within a 3000m circular buffer	1. Weekly frequency of: walking, running/jogging, biking<br><br>2. Total PA score (frequency and intensity of engaging in 13 specific activities)	self-reported instrument	No relevant results for green space	Fast food consumption and physical activity variables were not significantly associated with latent classes in fully-adjusted models (Model 3	age, study year, race, gender, study center, educational attainment, census tract-level education and income	N/A	N/A
Chong et al., 2019	men and women; ≥ 45 years; T2D (Australia)	3.3 (mean) years	Percent green space in 500m, 1000m, and 2000m road network buffer	1. Sitting (hrs/week)<br><br>2. Walking (min/week)<br><br>3. MVPA (min/week)	self-reported instrument	linear regression<br><br>XXX XXX(for the three outcomes)	mixed	age, gender, country of birth, education, disadvantage, physical functioning, BMI, psychological distress		
Josey and Moore., 2018	men and women; ≥ 25years; urban residents; (Canada)	5 years	Distance to parks and green spaces	Physically active/ inactive (*meet at least one criteria: (1) ≥ 3 days of vigorous activity of at least 20 min/ day, (2) ≥ 5 days of*	self-reported instrument	regression<br><br>X	no association between unadjusted models and PA; no results for adjusted	sex, age, self-reported health status, SES, household language,		

				moderate activity or walking of at least 30 min/ day, or (3) ≥ 5 or more days of any combination of walking, moderate or vigorous equalling to minimum of 600 MET-min/week)				marriage status, residential duration, wave		
Michael et al., 2010	men; ≥ 65 years; (USA)	3.6 (mean) years	distance to park	Maintenance or increase in frequency of walking (*min/ week*) (*Increased: 30 minutes or more, decreased: 30 minutes or more; or remained stable*)	self-reported instrument	logistic regression<br><br>Low SES: X<br>High SES: N (*shorter distance associated with higher probability of PA*)	distance associated with increase in PA in high SES but not in low	age, race education, occupation, marital status, self-reported health, BMI, smoking, drinking, chronic conditions	Restricting to men who did not change address between baseline and follow-up	no difference in results
Yang et al., 2017	men and women; ≥ 40-79 years; (United Kingdom)	7 years	Presence of park or green space in 800m circular buffer	Uptake and maintenance of active commuting (*Active commuter: those reporting 'always' or 'usually' travelling to work by bicycle or on foot*)	self-reported instrument	regression<br><br>X	no association but only univariate models assessed	no adjustment		
Cleland et al., 2009	women parents; mean: 42.4 years; (Australia)	2 years	amount of greenery and quality of parks, self-reported satisfaction	1. Walking for leisure (*low (less than 90min/week) or high (≥90 min/week)*)	self-reported instrument	Walking for leisure:<br>Maintenance: P (1.80) P (1.73)<br><br>Increase: X X	walking for leisure: amount of greenery was associated with persistently high walking (RR= 1.80 (1.04Ð3.13)). Satisfied with	Age, marital status, number of children in the household, highest level of schooling		

				2. Walking for transport (*low (less than 30 min/week) or high (≥30 min/week))*		Walking for transport:<br><br>Maintenance: Xx<br><br>Increase:xP(1.71)	quality of park was associated with persistently high walking. 1.73 (1.17Ð2.57). Walking for transportation: Those satisfied with quality of parks showed higher probability of increasing walking. 1.71 (1.06Ð2.75)			
Picavet et al., 2016	men and women; ≥ 18 to 55 years; (Netherlands)	15 years	total percent green space, agricultural green, urban green in 125m and 1000m circular buffer;	1. MVPA (h/week)<br><br>2. Sports<br><br>3. Bicycling,<br><br>4. Gardening<br><br>5. Odd jobs<br><br>*(frequency: min/week)*	self-reported instrument	1. Mixed<br>2. Mixed<br>3. Mixed<br>4. Mixed	Type of GS associated with type of PA and depression. Some association with aspects of HQoL.	age, sex, SES		
Hogendorf et al., 2020	men and women; mean: 53 years;	10 years	area of green space within a 1000m circular buffer;	1. Walking for leisure	self-reported instrument	Distance:<br>Area of GS: XXXX	No association with area; association	marital status, income, employment,		

	(Netherlands)		distance to green space	2. Walking for commute<br><br>3. Cycling for leisure<br><br>4. Cycling for leisure and commute<br><br>5. Total\<br><br>(*frequency, average number of days/ week, and hours and minutes/ day, spent walking and cycling as part of an active commute and for leisure purposes*)		Distance:<br>Total walking: P (decrease with distance)<br>Walking for commute: P<br>Walking for leisure N	with walking and distance	smoking, self-rated health		
Coogan et al., 2009	black women;<br>≥ 21-69 years;<br>(USA)	2-6 years<br><br>98,280 person-years of follow-up.	distance to park	1. Utilitarian walking<br><br>2. Exercise walking<br><br>(*frequency hours/ week)*	self-reported instrument	logistic regression<br><br>N for exercise walking<br>x for utilitarian	longer distance associated with less PA	Age, region, BMI, smoking, alcohol, marital status, parity, caregiver status, residential moves, chronic conditions, history of cancer, moving residence,	Adjustment for clustering at group block level	Similar results

								vacant housing, SES, crime		
Sugiyama et al., 2015	men and women; mean: 54.4 years; (Australia)	7 years	park or nature reserve in the neighbourhood, self-reported	1. Maintenance of regular walking (*Walking for recreation, exercise, and transport in the last week (frequency no. times/ week)*)	self-reported instrument	X for maintenance of regular walking<br><br>stratification by work status:<br><br>working: x<br>stopped working: x	No association between park and maintenance of regular walking.	Age, sex, education, work status change, child change, mobility, BMI		
Dalton et al., 2016 b	men and women; mean age at baseline 62.2	7.5 (mean) years	percent green space at baseline for non-movers; 800m,	1. Total<br>2. Outdoor<br>3. Recreational (*frequency MET h/ week*)	self-reported instrument	regression<br><br>PPP all three	decline of 8.0 MET h/wk for overall activity if they lived in the greenest neighbourhoods against a predicted decline of 12.1 MET h/wk for participants in the least green neighbourhoods.	age, sex, marital status, waist to hip ratio, BMI, morbidity, urban/ rural location	Repeating analysis using 3000m, 5000m circular buffers	Similar results
Faerstein et al., 2018	men and women; ≥ 18 years; civil servants; (Brazil)	13 years	NDVI (800m circular buffer); presence of trees (visual inspection); proximity to waterfronts;	Non-work physical activity (*yes/ no in past 4 weeks*)	self-reported instrument	regression<br><br>PPP	higher NDVI; trees and closer prox to waterfronts associated with more PA	Sex, race, education, income, neighbourhood contextual variables	Buffer sizes: 400m, 800m, 1200m and 1600m; further adjustment for season, time at residence	No difference in results

Lin et al., 2020	men and women; ≥ 65-98 years; (Hong Kong)	7.8 (mean) years	NDVI in 300m circular buffer	Total PA level (*frequency in number of days and hours/ week*)	self-reported instrument	Latent profile analysis/ Exposure grouped into classes with other built environment variables		age, sex, marital status, education level, alcohol consumption, smoking, living alone, self-rated health, chronic conditions, functional impairment	linear-effect model; excluding movers; excluding those with functional impairment; excluding those lost to follow-up	No substantial differences

Depression

Reference	Population Description	Exposure Type (green/ blue space)	Exposure Description	Outcome Description	Outcome Measure	Estimate Type	Effect Estimate	Confounders	Sensitivity Analyses	Sensitivity Results
Banay et al., 2019	women nurses; 30–55 years; (USA)	Green Space	NDVI averages for each year of follow-up; 250m and 1250m circular buffers	Depression	first self-report of physician/ clinician diagnosis of depression or new regular use of antidepressants	Hazard Ratio<br><br>NN	no significant association in an age-adjusted model; lower risk of incident depression in people living in greenest quintile compared to those of least green in both minimally-adjusted and fully adjusted models in both buffer sizes.	age, race, mental health, marital status, educational attainment, husband's educational attainment, population density, income, median home value, PM2:5 level, BMI, smoking status and pack-years of smoking, alcohol consumption, physical activity, physical function, bodily pain (baseline), social network strength, care to ill family members (baseline), difficulty sleeping (baseline)		
Gariepy et al., 2015	men and women; 18-80 years (Canada)	Green Space	Presence of a park within a 500m circular buffer	Depression (y/n)	self-reported instrument Composite International Diagnostic Interview	Latent class growth models (LCGM)<br><br>N	Point estimates suggest that the presence of parks was	age, sex, marital status, education, income adequacy, childhood life events, chronic	Restricting to non-movers and non-rural dwellers	Similar results

					Short-Form for Major Depression (CIDI-SFMD)		associated with a 95% lower odds of having a depression symptom episode for the group following a low probability trajectory of depression symptom episodes and a 26% lower odds for the group following moderate probability of depression symptom episodes (Table 2).	condition, family history of depression		
Tomita et al., 2017	men and women; mean 20 years (South Africa)	Green Space	NDVI as annual mean for years of follow-up	Depression (y/n)	self-reported instrument CES-D	Odds Ratio<br><br>X	No association between NDVI and depression in any adjusted model	age, sex, marital status, race, household income, employment, rurality		

Pun et al., 2018	men and women; ≥ 57-85 years; (USA)	Green Space	NDVI seasonal changes in 250m 1000m circular buffer	Depression; anxiety (continuous)	self-reported instrument 7-item Hospital Anxiety and Depression Scale – anxiety subscale (HADS-A), and 11-item Center for Epidemiological Studies – Depression (CESD-11) Scale	linear mixed model (beta coefficient) XX	No association; only in unadjusted	age, gender, questionnaire year, season, region, education attainment, 3-day moving average of temperature, 60- months moving average of $PM_{2.5}$	Restricted to: 1. Not taking antidepressants 2. living in metropolitan statistical area 3. 1000m circular buffer	consistent with primary analyses
Fernandez-Nino et al., 2019	men and women; ≥ 55 years; (Mexico)	green space	street trees; total length of street covered in trees in a 950m road network buffer	Depression (y/n)	self-report of doctor diagnosis	mixed effect model X	no association	sex, age, income index, functional limitations, margination index of the municipality	comparing individuals with and without complete information	some significant differences in education, SES, urban area
Gariepy et al., 2014	men and women; ≥ 18-80 years; diabetes (any type) (Canada)	Green space	NDVI and percent of neighbourhood with parks and recreational facilities in a 500m, 1000m, 1500m circular buffer	Depression (y/n)	self-reported instrument Patient Health Questionnaire (PHQ–9)	hazard ratio X	no association	sex, age, marital status, family income, educational level, employment	Buffer sizes: 500 m, 1000 m, 1500 m	consistent with primary analyses

Picavet et al., 2016	men and women; ≥ 18 to 55 years; (Netherlands)	green space	percent green space in 250m and 1000m circular buffer	Depression (y/n)	self-reported instrument CES-D	PA: NPX QOL: XPN Depression: NNpx	Type of GS associated with type of PA and depression. Some association with aspects of HQoL.	age, sex, SES		
Astell-Burt and Feng, 2019	men and women; ≥ 45 years; Australia	green space	total percent green space and tree canopy in a 1600m road network buffer	Depression or anxiety (y/n)	self-report of doctor diagnosed	regression x	no association	age, sex income, education, economic status, couple status		
Melis et al.,2015	men and women; ≥ 20-65 years; (Italy)	green space	availability of green space measured via index by area units	Depression (y/n).	antidepressant use	regression X	no association	sex, age, education level, activity status, citizenship, residential stability at same address		

Diabetes:

Reference	Population Description	Exposure Description	Outcome Description	Outcome Measure	Estimate Type	Effect Estimate	Confounders	Sensitivity Analyses	
Lee et al., 2017	men and women; ≥ 19 years (48.6 years mean) (USA)<br><br>Generation Three cohorts of the Framingham Heart Study	Percent green space within a census block	Diabetes (no mention of type)	Clinical samples and medication	Odds Ratio<br><br>X	no significant association for diabetes; no resulst reported for obesity	age, gender, smoking status, education, cohort status, fasting plasma glucose, BMI		
Paquet et al., 2014	men and women; ≥ 18 years (Australia)	NDVI in 1000m road network buffer	Diabetes (no mention)	Clinical samples	relative Risk<br>X	No association between greenness and any of the outcomes	age, gender, smoking status, education, cohort status, fasting plasma glucose, BMI		
Clark et al., 2017	men and women; ≥ 45-84 years; urban residents (Canada)	NDVI yearly and seasonal; in 100m circular buffer	Diabetes (no mention of type)	Medical records	regression<br><br>N	Greenness showed a protective association with the incidence of diabetes, with an IQR increase in greenness being associated with a 10% decrease in odds for the incidence of	gender, age, area-level household income, walkability, pollution	census measure of neighbourhoods with >10% of the population being of South Asian ethnicity. A similar analysis was conducted for Chinese ethnicity	after adjustment, the effect stayed the same for south Asian, but incidence of diabetes increased with increasing greenness in the Chinese ethnicity

						diabetes in the fully adjusted models			
Liao et al., 2019	pregnant women; 25-29 years mean age group (China)	NDVI for conception years; 300m circular buffer	Gestational diabetes	Clinical samples	GEE models<br><br>N	pregnant women who re- sided in higher quartiles of residential NDVI had lower risks of incident IGT and GDM. The adjusted RRs for incident IGT and GMD were 0.77 (95% CI: 0.62, 0.96) and 0.66 (95% CI: 0.52, 0.84) for pregnant women who resided in the highest quartile of NDVI	age, education years, BMI, passive smoking during pregnancy, parity, season of conception, income, urban areas	buffer size: 1000m; NDVI cell value of residential address	consistent with primary analyses
Dalton et al., 2016a	men and women;<br><br>≥ 40-80 years;<br><br>(United Kingdom)	percent green space; in 800m,	Diabetes (type 2)	Self-report of doc diagnosed or medication	hazard ratio<br><br>N	The hazard ratio remained similar (HR 0.81; 95% CI 0.65, 0.99; p = 0.042) after adjusting for age, sex, BMI,	sex, age, BMI, parental diabetes, SES	1. road network buffers<br>2. 3000m, 5000m circular buffer	1. small changes in effect size<br>2. no significant association

						whether a parent had been diag-nosed with diabetes and SES			
Astell-Burt and Feng, 2020	men and women; ≥ 45 years; Australia	Percent total green space and tree canopy in a 1600m road network buffer	Diabetes (no mention)	Self-report of doctor diagnosed	regression<br><br>T2D: NX	more tree canopy associated with less incidence of 3 diseases; no association with percentage green	age, sex income, education, economic status, couple status	categorical exposures	consistent with primary analyses
Renzi et al., 2018	men and women; ≥ 35 years; (Italy)	NDVI and LAI ; in a 300m circular buffer	Diabetes (type 2)	Medical records	regression<br><br>X	no association	SES, marital status, educational level, occupation, place of birth, sex.		

Obesity

Reference	Population Description	Exposure Type (green/ blue space)	Exposure Indicator	Outcome Description	Outcome Measure	Estimate Type	Effect Estimate	Confounding	Sensitivity analyses	Sensitivity Results
Lee et al., 2017	men and women; ≥ 19 years (48.6 years mean) (USA)<br><br>Generation Three cohorts of the Framingham Heart Study	Green Space	Percent green space within a census block	Obesity (abdominal)	objectively-measured BMI	Odds Ratio<br><br>X	no significant association for diabetes; no resulst reported for obesity	age, gender, smoking status, education, cohort status, fasting plasma glucose, BMI		
Paquet et al., 2014	men and women; ≥ 18 years (Australia)	Green Space	NDVI in 1000m road network buffer	Obesity	objectively-measured (waist circumference)	relative Risk<br>X X X	No association between greenness and any of the outcomes	age, gender, education, household income, area-level deprivation		
Persson et al., 2018	men and women, ≥ 35-65 years; (Sweden)	Green Space	NDVI; time-weighted in a 100m, 250m, 500m circular buffer	obesity	objective measures of BMI	incidence rate ratio<br><br>P (men 100m), N (500m), X	positive association between increased NDVI exposure and risk of overweight for men in the fully adjusted model in the 100 m bu er (IRR	age, alcohol consumption, tobacco use, psychological distress, shift work, aircraft noise, railway noise, distance to water		

							1.157, 95% CI 1.001; 1.338 per IQR) (Table 3). However, no other associations were observed between NDVI and incidence of overweight or obesity for any of the sexes or bu ers. With regard to central obesity, NDVI exposure was associated with a lower risk in both sexes and all bu ers, although, it was only statistically signi cant for women in the 500 m bu er (IRR			

							0.884, 95% CI 0.793; 0.986 per IQR).			
Halonen et al., 2014	men and women; public sector employees; mean: 47.7 years (SD 5 9.1) (non-movers) and among the movers 41.8 (SD 5 9.8)	green space and blue space	distance to (usable)green space and distance to blue space in meters, objectively measured	obesity and overweight	self-reported BMI	logistic regression<br><br>Blue Areas<br><br>Non-movers: XP (More overweight with longer distances)<br>Movers: XXXX<br><br>Green:<br>Movers:<br>P (increased)X X<br>non-movers:<br>P (overweight) X	longer distances associated with more overweight for both green and blue areas<br><br>Movers:<br>Ref: (remained close)<br>Decreased[d]<br>Increased[e]<br>Remained distant[f]<br><br>non-movers: distance	age, sex, education, chronic disease, neighbourhood socioeconomic disadvantage, BMI, smoking, heavy alcohol, physical inactivity		
Picavet et al., 2016	men and women; ≥ 18 to 55 years; (Netherlands)	green space	percent green space in 125m and 1000m circular buffer; change of total, agricultural and urban green	Overweight and Obesity	objectively-measured BMI	XX(P for change in total green)xx (for change in type of green)	Type of GS associated with type of PA and depression. Some association with aspects of HQoL.	age, sex, SES		

Hobbs et al., 2019	men and women; ≥18-89 years; (United Kingdom)	green space	presence of park in a 2000m circular buffer	obesity	BMI, self-reported	regression<br><br>X	no association	age, sex, education, deprivation, population density	Buffer sizes: 1600m, 2000m	

Reference	Population Description	Exposure Type (green/ blue space)	Exposure Indicator	Outcome Description	Outcome Measure	Estimate Type	Effect Estimate	Confounders	Sensitivity Analyses	Sensitivity results
Dalton and Jones, 2020	men and women; mean 59.2 years; (United Kingdom)	Green Space	percent green space at baseline in a 800m circular buffer	CVD (ischaemic heart disease or cerebro-vascular disease ICD-10)	health register	incidence rate/ hazard ratio<br><br>N	Annual rates of incident CVD increased from 29.2 per 10,000 person-years (1 in 342) for those aged 40–49, to 1198.4 per 10,000 person years (1 in 8.3) for those aged 80–90 years. Adjusted HR: 0.97	sex, age, BMI, diabetes, SES (individual and neighbourhood)	1. non-movers<br><br>2. buffer size (3000m, 5000m) and road network buffers	1. consistent with primary analyses<br><br>2. consistent with primary analyses
Tamosiunas et al., 2014	men and women; ≥ 45-72 years; (Lithuania)	Green Space	Distance to park and park use self-reported	CVD (MI, ischemic changes, angina, ECG findings)	Health register	P for non-users	for CVD (and only for non-users), no reported for obesity	age, sex, education, smoking, arterial hypertension, physical activity, total cholesterol level, fasting glucose level, BMI, diabetes mellitus, cognitive function, symptoms of		

								depression, self-rated health, and quality of life		
Astell-Burt and Feng, 2020	men and women; ≥ 45 years; Australia	Green space	Percent green space and tree canopy in a 1600m road network buffer	CVD (no mention)	self-report of doctor diagnosed	regression<br><br>CVD: NX	more tree canopy associated with less incidence of 3 diseases; no association with percentage green	age, sex income, education, economic status, couple status	categorical exposures	consistent with primary analyses

Reference	Population Description	Exposure Type (green/ blue space)	Exposure Indicator	Outcome Description	Outcome Measure	Estimate Type	Effect Estimate	Confounding	Sensitivity Analyses	Sensitivity Results
Datzman et al.,	men and women; mean 49.33 years;<br><br>(Germany)	Green Space	NDVI; average for 4 years for postcode districts	colorectal; mouth and throat, prostate, breast; non-melanoma skin	health record	passion regression<br><br>Colorectal: x<br>mouth: N<br>skin: N<br>prostate: x<br>Breast: N	An increase in NDVI by 10% revealed associations with mouth and throat cancer with an 11% decrease in RR and with NMSC with a 16% de- crease in RR. No associations were found for prostate or breast cancer and NDVI. Colorectal cancer was not affected by any of the exposures)	age, sex, alcohol-related disorder, absolute number of physician contacts, proportion of short and long-term unemployment	Excluding movers	consistent with primary analyses

Conroy et al., 2017	women; ≥ 45-75 years; (African Americans, Japanese Americans, Latinos, Native Hawaiians, and Whites) (USA)	Green Space	Presence of a park; (number in a residential block group)	Invasive breast cancer (Tumour characteristics)	health register	hazard ratio<br><br>X	No significant association found between risk of cancer and presence of a park in the area, that goes for all analyses, even those stratified by race.	age, clustering effect of block group, ethnicity, risk factors (family history of BC, age at menarche, age at first live birth, number of children, hormone replacement therapy, ever had mammography, alcohol use, physical activity, education), baseline BMI and adult weight change, neighbourhood SES and all neighbourhood obesogenic factors	Addition of frailty models	No results given
Haraldsdottir et al., 2017	women; mean: 53.9 years<br><br>(Iceland)	blue space	coastal residence, self-reported	breast cancer	health registers	hazard ratio<br><br>N	.Compared to women born and raised in the capital area, early life residence in coastal villages weakly associated with a lower risk of breast cancer diagnosis	age, birth cohort, education, physical activity, parity, height, BMI in midlife, age at menarche, age at first child	repeating analyses with imputed missing values for some confounders	very slight attenuation of effect estimate

Stroke:

Reference	Population Description	Exposure Type (green/ blue space)	Exposure Indicator	Outcome Description	Outcome Measure	Estimate Type	Effect Estimate	Confounders	Sensitivity Analysed	Sensitivity Results
Paul et al., 2020	men and women; ≥ 35-100 years; urban residents Ontario (Canada)	Green Space	NDVI annual values in a250m circular buffer	Stroke	health register	Hazard Ratio<br><br>N	In all models incrementally adjusting for covariates, green space was associated with reduced incidence of both dementia and stroke For stroke, we found a HR of 0.96 (0.95–0.98) per IQR increase.	age, sex, SES, comorbidities, northern residence, population density, air pollution	1. categorical exposure<br>2. adjustment for deprivation and neurologists count<br>3. GS at a larger buffer 500m<br>4. indirect adjustment for confounders	All consistent with primary analyses
Orioli et al., 2019	men and women; ≥ 30 years<br><br>(Italy)	Green Space	NDVI and LAI average for 2015 in 300m and 1000m circular buffer	Stroke	health register	hazard ratio<br><br>N	Lower risk of stroke in those living in highest quintile of Lai and NDVI compared to those of lowest (applicable for both buffers).	age, sex, educational level, marital status, occupational status, place of birth, area-level socioeconomic position.	1. smoking<br>2. frailty; correlation between baseline and follow-up NDVI<br>3. green space categorical variables	???

							LAI 300: 0.977<br>LAI 1000: 0.982<br>NDVI 300: 0.976<br>NDVI 1000: 0.981			

Frailty

Reference	Population Description	Exposure Type (green/ blue space)	Exposure Indicator	Outcome Description	Outcome Measure	Estimate Type	Effect Estimate	Confounders	Sensitivity Analyses	Sensitivity Results
Yu et al.,	men and women; ≥ 65 years; Hong Kong	Green Space	NDVI at baseline in a 300m circular buffer	Frailty	self-reported instrument 5-item Cardiovascular Health Study<br><br>frailty phenotype	logistic regression<br><br>P (indicates improvement in frailty status)	frailty status of participants living in neighborhoods with more than 34.1% green space (the highest quartile) at baseline was more likely to improve at the 2-year follow-up than it was for those living in neighborhoods with 0 to 4.5% (the lowest quartile) (OR: 1.29, 95% CI: 1.04-1.60). no association by sex	age, sex, marital status, SES, current smoking status, alcohol intake, diet quality, baseline frailty status, number of diseases, cognitive function, physical activity, depression		
Zhu et al., 2020	men and women; ≥ 65 years; (China)	green space	NDVI; annual averages for each year in	frailty	self-reported instrument 39 self-reported items	mixed effect model<br>P (improvement)	. In the fully adjusted regression, each 0.1-unit in- crease in	age, sex, ethnicity, marital status, geographic region, urban or rural		

			500m buffer				annual average NDVI was related to a 2% higher odds of improvement in the frailty status (OR: 1.02, 95% CI: 1.00, 1.04), h 0.1-unit increase in annual average NDVI was associated with an OR of 0.96 (95% CI: 0.93, 0.99) of frailty.	residence, education, occupation, financial support, social and leisure activity, smoking status, drinking status, physical activity		

Reference	Population Description	Exposure Type (green/ blue space)	Exposure Indicator	Outcome Description	Outcome Measure	Estimate Type	Effect Estimate	Confounders	Sensitivity Analyses	Sensitivity Results
Paquet et al., 2014	men and women; ≥ 18 years (Australia)	Green Space	NDVI in 1000m road network buffer	Hypertension	clinical samples: diastolic/systolic BP 85/ 130 mmHg or treatment for hypertension	relative Risk X	No association between greenness and any of the outcomes	age, gender, education, household income, area-level deprivation		
Picavet et al., 2016	men and women; ≥ 18 to 55 years; (Netherlands)	green space	percent green space in 250m and 1000m circular buffer	Hypertension	Clinical measures: diastolic/systolic BP 85/ 130 mmHg	X for all	Type of GS associated with type of PA and depression. Some association with aspects of HQoL.	age, sex, SES		
Astell-Burt and Feng, 2020	men and women; ≥ 45 years; Australia	Green space	Percent green space and tree canopy in a 1600m road network buffer	Hypertension	self-report of doctor diagnosed	Hypertension NX	more tree canopy associated with less incidence of 3 diseases; no association with percentage green	age, sex income, education, economic status, couple status	categorical exposures	results consistent with primary analyses

Reference	Population Description	Exposure Indicator	Outcome Description	Outcome Measure	Estimate Type	Confounders	Sensitivity Analyses	Sensitivity Results
Paul et al., 2020	men and women; ≥ 35-100 years; urban residents of Ontario (Canada)	NDVI (*250m circular buffer*)	Stroke	Health register	N	age, sex, SES, comorbidities, northern residence, population density, air pollution	Repeating analysis: 1.categorical exposure 2. adjustment for deprivation and neurologists count 3. 500m circular buffer 4. indirect adjustment for confounders	All consistent with primary analyses
Orioli et al., 2019	men and women;	NDVI	Stroke	Health register		age, sex, educational level, marital status,	Repeating analysis:	???

	≥ 30 years<br><br>(Italy)	LAI<br><br>(*300m,1000m circular buffer*)			N (NDVI, 300m)<br>N (NDVI, 1000m)<br>N (LAI, 300m)<br>N (LAI, 1000m)	occupational status, place of birth, area-level socioeconomic position.	1. adjusting for smoking<br>2. frailty; correlation between baseline and follow-up NDVI<br>3. green space categorical variables	
Yu et al., 2018	men and women;<br>≥ 65 years<br>(Hong Kong)	NDVI<br><br>(*300m circular buffer*)	Frailty	Self-reported instrument (5-item Cardiovascular Health Study)<br><br>Frailty Phenotype	P (indicates improvement in frailty status)	age, sex, marital status, SES, current smoking status, alcohol intake, diet quality, baseline frailty status, number of diseases, cognitive function, physical activity, depression		
Zhu et al., 2020	men and women;<br>≥ 65 years<br>(China)	NDVI<br><br>(500m buffer)	Frailty	Self-reported instrument<br><br>(39 item self-reported items)<br><br>Frailty Index	P (indicates improvement in frailty status)	age, sex, ethnicity, marital status, geographic region, urban or rural residence, education, occupation, financial support, social and leisure activity, smoking status, drinking status, physical activity		
Chang et al., 2019	men and women<br>mean age: 43.36 (20.44) years<br><br>(Taiwan)	NDVI<br><br>(*2000m circular buffer around hospital*)	Schizophrenia	Physician-diagnosed	N	age, sex, health insurance rate, classification of the insured, temperature, relative humidity, precipitation	Repeating analysis using:<br>1. NDVI as continuous variable in buffer sizes: 1000, 1250, 1500, 1750, 2000;	Lower HR for increasing buffer sizes; low HR with increasing greenness in those with low

							2. combined effect of greenness and PM10<br><br>Restricting analyses to<br>1. urban residence<br>2. health insurance rate<br>3. sex	insurance rate; lower HR in men who live in greener areas; no association in women
Paquet et al., 2014	men and women; ≥ 18 years (Australia)	NDVI<br><br>(*1000m road network buffer*)	Dyslipidaemia	Clinical samples	X	age, gender, education, household income, area-level deprivation		
de Keijzer et al., 2019a	men and women; ≥ 35-55 years civil servants (United Kingdom)	NDVI<br><br>VCF (*500m, 1000m circular buffer, LSOA*)	Metabolic Syndrome	Clinical Samples	N (NDVI, 500m)<br>X (NDVI, 1000m)<br>X (NDVI, LSOA)<br><br>N (VCF, 500m)<br>N (VCF ,1000m)<br>N (VCF , LSOA)	age, sex, ethnicity, individual socioeconomic status (education and employment grade), neighbourhood socioeconomic status (income and employment deprivation).	Restricted analysis to:<br>1. non-movers; urban areas<br>2. non-white ethnicity<br>3. participants living in England	many associations with VCF, all HRs below 1; no association between summer NDVI; similar to full models
Meyer et al., 2015	men and women; ≥ 18-30 years;	Number of parks	Diet Quality	Self-reported instrument	No relevant results	age, study year, race, gender, study center, educational attainment, census tract-level education and income		

	black and white<br>(USA)	(*3000m circular buffer*)						
Pun et al., 2018	men and women;<br>≥ 57-85 years<br><br>(USA)	NDVI<br><br>(*1000m circular buffer*)	Anxiety	Self-reported instrument	X			
de Keijzer et al., 2019b	men and women;<br>≥ 35-55;<br>civil servants<br><br>(United Kingdom)	NDVI<br><br>EVI<br><br>(*500m, 1000m circular buffer*)<br><br>Distance to blue space (*any visible water*)<br><br>Distance to green or blue space	Physical Functioning<br><br>1. Walking Speed<br><br>2. Grip Strength	Clinical measures	1. Walking Speed<br><br>P (all NDVI & EVI)<br><br>X (all distance to blue space, distance to green space; distance to green 7 blue space)<br><br>2. Grip Strength<br><br>X (all EVI)<br>P (all NDVI)<br>X (all distance to blue space, distance to	sex, ethnicity, marital status, height, alcohol use, intake of fruit and vegetables, smoking, rurality, education, employment grade, Index of Multiple Deprivation (IMD), income score and of the IMD, employment score	sensitivity: Repeating analysis using: imputed data for the missing covariate values<br>1. including only observations from England to test the influence of country (England, Scotland, Wales),<br>2. excluding non-white participants to test the influence of ethnicity<br>3.excluding rural areas<br>1. excluding participants who	

					green space; distance to green 7 blue space		changed postcode in the study period to test the influence of moving and 2. excluding participants with limitations in walking over one mile at baseline	

de Keijzer et al., 2019b	men and women;<br>≥ 35-55;<br>civil servants<br><br>(United Kingdom)	NDVI<br><br>EVI<br><br>(*500m, 1000m circular buffer*)<br><br>Distance to blue space (*any visible water*)<br><br>Distance to green or blue space	Physical Functioning<br><br>1. Walking Speed<br><br>2. Grip Strength	Clinical measures	1. Walking Speed<br><br>P (all NDVI & EVI)<br><br>X (all distance to blue space, distance to green space; distance to green 7 blue space)<br><br>2. Grip Strength<br><br>X (all EVI)<br>P (all NDVI)<br>X (all distance to blue space, distance to green space; distance to green 7 blue space	sex, ethnicity, marital status, height, alcohol use, intake of fruit and vegetables, smoking, rurality, education, employment grade, Index of Multiple Deprivation (IMD), income score and of the IMD, employment score	sensitivity: Repeating analysis using: imputed data for the missing covariate values<br>1. including only observations from England to test the influence of country (England, Scotland, Wales),<br>2. excluding non-white participants to test the influence of ethnicity<br>3.excluding rural areas<br>1. excluding participants who changed postcode in the study	

							period to test the influence of moving and 2. excluding participants with limitations in walking over one mile at baseline	
Yuchi et al., 2020	men and women;<br>≥ 45-84 years (Canada)	NDVI<br><br>(*100m circular buffer*)	1. Parkinson's disease<br>2. Multiple sclerosis	Medical records	1. X<br>2. X	Parkinson's disease: Age, sex, comorbidities, household income, education, ethnicity<br><br>Multiple sclerosis: Age, sex, comorbidities, household income, education and ethnicity, comorbidities, household income, education, ethnicity		

Picavet et al., 2016	men and women;<br>≥ 18-55 years (Netherlands)	Percent total green space<br><br>Percent agricultural green<br><br>Percent urban green<br><br>(*125m, 1000m circular buffers*)	Quality of Life<br><br>Component of SF-36:<br><br>1. Physical Functioning<br>2. Role Limitation (physical)<br>3. Pain<br>4. General Health<br>5. Vitality<br>6. Social Functioning<br>7. Role Limitation (mental)	Self-reported instrument (Short Form, 36 (SF-36))	X (all Pain, General health, Vitality, Social Functioning, Mental Role limitation)<br><br>Role Limitation (physical):<br>P (agricultural green)<br>P (all total green)<br>X (all other)<br><br>Physical Functioning:<br><br>N (urban green)<br>P (total green, 1000m)<br><br>X (all other)	age, sex, SES		

de Keijzer et al., 2019b	men and women;<br>≥ 35-55;<br>civil servants<br><br>(United Kingdom)	NDVI<br><br>EVI<br><br>(*500m, 1000m circular buffer*)<br><br>Distance to blue space (*any visible water*)<br><br>Distance to green or blue space	Physical Functioning<br><br>1. Walking Speed<br><br>2. Grip Strength	Clinical measures	1. Walking Speed<br><br>P (all NDVI & EVI)<br><br>X (all distance to blue space, distance to green space; distance to green 7 blue space)<br><br>2. Grip Strength<br><br>X (all EVI)<br>P (all NDVI)<br>X (all distance to blue space, distance to green space; distance to green 7 blue space	sex, ethnicity, marital status, height, alcohol use, intake of fruit and vegetables, smoking, rurality, education, employment grade, Index of Multiple Deprivation (IMD), income score and of the IMD, employment score	sensitivity:<br><br>Repeating analysis using:<br>imputed data for the missing covariate values<br><br>1. including only observations from England to test the influence of country (England, Scotland, Wales),<br><br>2. excluding non-white participants to test the influence of ethnicity<br><br>3.excluding rural areas<br><br>1. excluding participants who changed postcode in the study period to test the influence of moving and 2. excluding participants with limitations in walking over one mile at baseline	
Yuchi et al., 2020	men and women;	NDVI	1. Parkinson's disease	Medical records	1. X<br>2. X	Parkinson's disease: Age, sex,		

	≥ 45-84 years (Canada)	(*100m circular buffer*)	2. Multiple sclerosis			comorbidities, household income, education, ethnicity<br><br>Multiple sclerosis: Age, sex, comorbidities, household income, education and ethnicity, comorbidities, household income, education, ethnicity		
Picavet et al., 2016	men and women;<br>≥ 18-55 years (Netherlands)	Percent total green space<br><br>Percent agricultural green<br><br>Percent urban green<br><br>(*125m, 1000m circular buffers*)	Quality of Life<br><br>Component of SF-36:<br><br>1. Physical Functioning<br>2. Role Limitation (physical)<br>3. Pain<br>4. General Health<br>5. Vitality	Self-reported instrument (Short Form, 36 (SF-36))	X (all Pain, General health, Vitality, Social Functioning, Mental Role limitation)<br><br>Role Limitation (physical):<br>P (agricultural green)	age, sex, SES		

			6. Social Functioning<br>7. Role Limitation (mental)		P (all total green)<br>X (all other)<br><br>Physical Functioning:<br><br>N (urban green)<br>P (total green, 1000m)<br><br>X (all other)			

Chang et al., 2019	men and women mean age: 43.36 (20.44) years<br><br>(Taiwan)	NDVI<br><br>(*2000m circular buffer around hospital*)	Schizophrenia	Physician-diagnosed	N	age, sex, health insurance rate, classification of the insured, temperature, relative humidity, precipitation	Repeating analysis using:<br>1. NDVI as continuous variable in buffer sizes: 1000, 1250, 1500, 1750, 2000;<br><br>2. combined effect of greenness and PM10<br><br>Restricting analyses to<br>1. urban residence 2. health insurance rate<br>3. sex	Lower HR for increasing buffer sizes; low HR with increasing greenness in those with low insurance rate; lower HR in men who live in greener areas; no association in women
Paquet et al., 2014	men and women;<br>≥ 18 years (Australia)	NDVI<br><br>(*1000m road network buffer*)	Dyslipidaemia	Clinical samples	X	age, gender, education, household income, area-level deprivation		
de Keijzer et al., 2019a	men and women;<br>≥ 35-55 years civil servants	NDVI<br><br>VCF (*500m, 1000m*	Metabolic Syndrome	Clinical Samples	N (NDVI, 500m)<br>X (NDVI, 1000m)	age, sex, ethnicity, individual socioeconomic	Restricted analysis to:	many associations with VCF, all HRs below 1;

	(United Kingdom)	*circular buffer, LSOA*)			X (NDVI, LSOA)<br><br>N (VCF, 500m)<br>N (VCF ,1000m)<br>N (VCF , LSOA)	status (education and employment grade), neighbourhood socioeconomic status (income and employment deprivation).	1. non-movers; urban areas<br>2. non-white ethnicity<br>3. participants living in England	no association between summer NDVI; similar to full models
Meyer et al., 2015	men and women;<br>≥ 18-30 years;<br>black and white<br>(USA)	Number of parks<br><br>(*3000m circular buffer*)	Diet Quality	Self-reported instrument	No relevant results	age, study year, race, gender, study center, educational attainment, census tract-level education and income		
Pun et al., 2018	men and women;<br>≥ 57-85 years<br><br>(USA)	NDVI<br><br>(*1000m circular buffer*)	Anxiety	Self-reported instrument	X			
de Keijzer et al., 2019b	men and women;<br>≥ 35-55;<br>civil servants<br><br>(United Kingdom)	NDVI<br><br>EVI<br><br>(*500m, 1000m circular buffer*)	Physical Functioning<br><br>1. Walking Speed<br><br>2. Grip Strength	Clinical measures	1. Walking Speed<br><br>P (all NDVI & EVI)<br><br>X (all distance to blue space,	sex, ethnicity, marital status, height, alcohol use, intake of fruit and vegetables, smoking, rurality, education,	sensitivity:<br><br>Repeating analysis using:<br>imputed data for the missing covariate values<br><br>1. including only observations	

		Distance to blue space (*any visible water*)<br><br>Distance to green or blue space			distance to green space; distance to green 7 blue space)<br><br>2. Grip Strength<br><br>X (all EVI)<br>P (all NDVI)<br>X (all distance to blue space, distance to green space; distance to green 7 blue space	employment grade, Index of Multiple Deprivation (IMD), income score and of the IMD, employment score	from England to test the influence of country (England, Scotland, Wales),<br><br>2. excluding non-white participants to test the influence of ethnicity<br><br>3.excluding rural areas<br><br>1. excluding participants who changed postcode in the study period to test the influence of moving and 2. excluding participants with limitations in walking over one mile at baseline	
Yuchi et al., 2020	men and women;<br>≥ 45-84 years (Canada)	NDVI<br><br>(*100m circular buffer*)	1. Parkinson's disease<br>2. Multiple sclerosis	Medical records	1. X<br>2. X	Parkinson's disease: Age, sex, comorbidities, household income, education, ethnicity<br><br>Multiple sclerosis: Age, sex, comorbidities,		

						household income, education and ethnicity, comorbidities, household income, education, ethnicity		
Picavet et al., 2016	men and women; ≥ 18-55 years (Netherlands)	Percent total green space<br><br>Percent agricultural green<br><br>Percent urban green<br><br>(*125m, 1000m circular buffers*)	Quality of Life<br><br>Component of SF-36:<br><br>1. Physical Functioning<br>2. Role Limitation (physical)<br>3. Pain<br>4. General Health<br>5. Vitality<br>6. Social Functioning<br>7. Role Limitation (mental)	Self-reported instrument (Short Form, 36 (SF-36))	X (all Pain, General health, Vitality, Social Functioning, Mental Role limitation)<br><br>Role Limitation (physical):<br>P (agricultural green)<br>P (all total green)<br>X (all other)<br><br>Physical Functioning:<br><br>N (urban green)	age, sex, SES		

					P (total green, 1000m)<br><br>X (all other)			

Chang et al., 2019	men and women mean age: 43.36 (20.44) years<br><br>(Taiwan)	NDVI<br><br>(*2000m circular buffer around hospital*)	Schizophrenia	Physician-diagnosed	N	age, sex, health insurance rate, classification of the insured, temperature, relative humidity, precipitation	Repeating analysis using:<br>1. NDVI as continuous variable in buffer sizes: 1000, 1250, 1500, 1750, 2000;<br><br>2. combined effect of greenness and PM10<br><br>Restricting analyses to<br>1. urban residence 2. health insurance rate<br>3. sex	Lower HR for increasing buffer sizes; low HR with increasing greenness in those with low insurance rate; lower HR in men who live in greener areas; no association in women
Paquet et al., 2014	men and women;<br>≥ 18 years (Australia)	NDVI<br><br>(*1000m road network buffer*)	Dyslipidaemia	Clinical samples	X	age, gender, education, household income, area-level deprivation		
de Keijzer et al., 2019a	men and women;<br>≥ 35-55 years civil servants	NDVI<br><br>VCF (*500m, 1000m*	Metabolic Syndrome	Clinical Samples	N (NDVI, 500m)<br>X (NDVI, 1000m)	age, sex, ethnicity, individual socioeconomic	Restricted analysis to:	many associations with VCF, all HRs below 1;

	(United Kingdom)	*circular buffer, LSOA*)			X (NDVI, LSOA)<br><br>N (VCF, 500m)<br>N (VCF ,1000m)<br>N (VCF , LSOA)	status (education and employment grade), neighbourhood socioeconomic status (income and employment deprivation).	1. non-movers; urban areas<br>2. non-white ethnicity<br>3. participants living in England	no association between summer NDVI; similar to full models
Meyer et al., 2015	men and women;<br>≥ 18-30 years;<br>black and white<br>(USA)	Number of parks<br><br>(*3000m circular buffer*)	Diet Quality	Self-reported instrument	No relevant results	age, study year, race, gender, study center, educational attainment, census tract-level education and income		
Pun et al., 2018	men and women;<br>≥ 57-85 years<br><br>(USA)	NDVI<br><br>(*1000m circular buffer*)	Anxiety	Self-reported instrument	X			
de Keijzer et al., 2019b	men and women;<br>≥ 35-55;<br>civil servants<br><br>(United Kingdom)	NDVI<br><br>EVI<br><br>(*500m, 1000m circular buffer*)	Physical Functioning<br><br>1. Walking Speed<br><br>2. Grip Strength	Clinical measures	1. Walking Speed<br><br>P (all NDVI & EVI)<br><br>X (all distance to blue space,	sex, ethnicity, marital status, height, alcohol use, intake of fruit and vegetables, smoking, rurality, education,	sensitivity:<br><br>Repeating analysis using:<br>imputed data for the missing covariate values<br><br>1. including only observations	

		Distance to blue space (*any visible water*)<br><br>Distance to green or blue space			distance to green space; distance to green 7 blue space)<br><br>2. Grip Strength<br><br>X (all EVI)<br>P (all NDVI)<br>X (all distance to blue space, distance to green space; distance to green 7 blue space	employment grade, Index of Multiple Deprivation (IMD), income score and of the IMD, employment score	from England to test the influence of country (England, Scotland, Wales),<br><br>2. excluding non-white participants to test the influence of ethnicity<br><br>3.excluding rural areas<br><br>1. excluding participants who changed postcode in the study period to test the influence of moving and 2. excluding participants with limitations in walking over one mile at baseline	
Yuchi et al., 2020	men and women;<br>≥ 45-84 years (Canada)	NDVI<br><br>(*100m circular buffer*)	1. Parkinson's disease<br>2. Multiple sclerosis	Medical records	1. X<br>2. X	Parkinson's disease: Age, sex, comorbidities, household income, education, ethnicity<br><br>Multiple sclerosis: Age, sex, comorbidities,		

						household income, education and ethnicity, comorbidities, household income, education, ethnicity		
Picavet et al., 2016	men and women; ≥ 18-55 years (Netherlands)	Percent total green space<br><br>Percent agricultural green<br><br>Percent urban green<br><br>(*125m, 1000m circular buffers*)	Quality of Life<br><br>Component of SF-36:<br><br>1. Physical Functioning<br>2. Role Limitation (physical)<br>3. Pain<br>4. General Health<br>5. Vitality<br>6. Social Functioning<br>7. Role Limitation (mental)	Self-reported instrument (Short Form, 36 (SF-36))	X (all Pain, General health, Vitality, Social Functioning, Mental Role limitation)<br><br>Role Limitation (physical):<br>P (agricultural green)<br>P (all total green)<br>X (all other)<br><br>Physical Functioning:<br><br>N (urban green)	age, sex, SES		

					P (total green, 1000m)<br><br>X (all other)			

Chang et al., 2019	men and women mean age: 43.36 (20.44) years<br><br>(Taiwan)	NDVI<br><br>(*2000m circular buffer around hospital*)	Schizophrenia	Physician-diagnosed	N	age, sex, health insurance rate, classification of the insured, temperature, relative humidity, precipitation	Repeating analysis using:<br>1. NDVI as continuous variable in buffer sizes: 1000, 1250, 1500, 1750, 2000;<br><br>2. combined effect of greenness and PM10<br><br>Restricting analyses to<br>1. urban residence 2. health insurance rate<br>3. sex	Lower HR for increasing buffer sizes; low HR with increasing greenness in those with low insurance rate; lower HR in men who live in greener areas; no association in women
Paquet et al., 2014	men and women;<br>≥ 18 years (Australia)	NDVI<br><br>(*1000m road network buffer*)	Dyslipidaemia	Clinical samples	X	age, gender, education, household income, area-level deprivation		
de Keijzer et al., 2019a	men and women;<br>≥ 35-55 years civil servants	NDVI<br><br>VCF (*500m, 1000m*	Metabolic Syndrome	Clinical Samples	N (NDVI, 500m)<br>X (NDVI, 1000m)	age, sex, ethnicity, individual socioeconomic	Restricted analysis to:	many associations with VCF, all HRs below 1;

	(United Kingdom)	*circular buffer, LSOA*)			X (NDVI, LSOA)<br><br>N (VCF, 500m)<br>N (VCF ,1000m)<br>N (VCF , LSOA)	status (education and employment grade), neighbourhood socioeconomic status (income and employment deprivation).	1. non-movers; urban areas<br>2. non-white ethnicity<br>3. participants living in England	no association between summer NDVI; similar to full models
Meyer et al., 2015	men and women;<br>≥ 18-30 years; black and white<br>(USA)	Number of parks<br><br>(*3000m circular buffer*)	Diet Quality	Self-reported instrument	No relevant results	age, study year, race, gender, study center, educational attainment, census tract-level education and income		
Pun et al., 2018	men and women;<br>≥ 57-85 years<br><br>(USA)	NDVI<br><br>(*1000m circular buffer*)	Anxiety	Self-reported instrument	X			
de Keijzer et al., 2019b	men and women;<br>≥ 35-55;<br>civil servants<br><br>(United Kingdom)	NDVI<br><br>EVI<br><br>(*500m, 1000m circular buffer*)	Physical Functioning<br><br>1. Walking Speed<br><br>2. Grip Strength	Clinical measures	1. Walking Speed<br><br>P (all NDVI & EVI)<br><br>X (all distance to blue space,	sex, ethnicity, marital status, height, alcohol use, intake of fruit and vegetables, smoking, rurality, education,	sensitivity:<br><br>Repeating analysis using:<br>imputed data for the missing covariate values<br><br>1. including only observations	

		Distance to blue space (*any visible water*)<br><br>Distance to green or blue space			distance to green space; distance to green 7 blue space)<br><br>2. Grip Strength<br><br>X (all EVI)<br>P (all NDVI)<br>X (all distance to blue space, distance to green space; distance to green 7 blue space	employment grade, Index of Multiple Deprivation (IMD), income score and of the IMD, employment score	from England to test the influence of country (England, Scotland, Wales),<br><br>2. excluding non-white participants to test the influence of ethnicity<br><br>3.excluding rural areas<br><br>1. excluding participants who changed postcode in the study period to test the influence of moving and 2. excluding participants with limitations in walking over one mile at baseline	
Yuchi et al., 2020	men and women;<br>≥ 45-84 years (Canada)	NDVI<br><br>(*100m circular buffer*)	1. Parkinson's disease<br>2. Multiple sclerosis	Medical records	1. X<br>2. X	Parkinson's disease: Age, sex, comorbidities, household income, education, ethnicity<br><br>Multiple sclerosis: Age, sex, comorbidities,		

						household income, education and ethnicity, comorbidities, household income, education, ethnicity		
Picavet et al., 2016	men and women;<br>≥ 18-55 years (Netherlands)	Percent total green space<br><br>Percent agricultural green<br><br>Percent urban green<br><br>(*125m, 1000m circular buffers*)	Quality of Life<br><br>Component of SF-36:<br><br>1. Physical Functioning<br>2. Role Limitation (physical)<br>3. Pain<br>4. General Health<br>5. Vitality<br>6. Social Functioning<br>7. Role Limitation (mental)	Self-reported instrument (Short Form, 36 (SF-36))	X (all Pain, General health, Vitality, Social Functioning, Mental Role limitation)<br><br>Role Limitation (physical):<br>P (agricultural green)<br>P (all total green)<br>X (all other)<br><br>Physical Functioning:<br><br>N (urban green)	age, sex, SES		

					P (total green, 1000m)<br><br>X (all other)			
